# Enhanced photoreduction of water catalyzed by a cucurbit[8]uril-secured platinum dimer†

**DOI:** 10.1039/d1sc03743a

**Published:** 2021-11-04

**Authors:** Ramin Rabbani, Sima Saeedi, Md Nazimuddin, Héctor Barbero, Nathalie Kyritsakas, Travis A. White, Eric Masson

**Affiliations:** Department of Chemistry and Biochemistry, Ohio University Athens Ohio 45701 USA masson@ohio.edu; GIR MIOMeT, IU CINQUIMA/Química Inorgánica, Facultad de Ciencias, Universidad de Valladolid Valladolid E47011 Spain; Molecular Tectonics Laboratory, University of Strasbourg, UMR UDS-CNRS 7140, Institut le Bel F-67000 Strasbourg France

## Abstract

A cucurbit[8]uril (CB[8])-secured platinum terpyridyl chloride dimer was used as a photosensitizer and hydrogen-evolving catalyst for the photoreduction of water. Volumes of produced hydrogen were up to 25 and 6 times larger than those obtained with the corresponding free and cucurbit[7]uril-bound platinum monomer, respectively, at equal Pt concentration. The thermodynamics of the proton-coupled electron transfer from the Pt(ii)–Pt(ii) dimer to the corresponding Pt(ii)–Pt(iii)–H hydride key intermediate, as quantified by density functional theory, suggest that CB[8] secures the Pt(ii)–Pt(ii) dimer in a particularly reactive conformation that promotes hydrogen formation.

## Abstract 2

The cucurbit[8]uril macrocycle can secure a platinum terpyridyl complex into a particularly reactive dimer that catalyzes the photoreduction of water.

## Graphical abstract

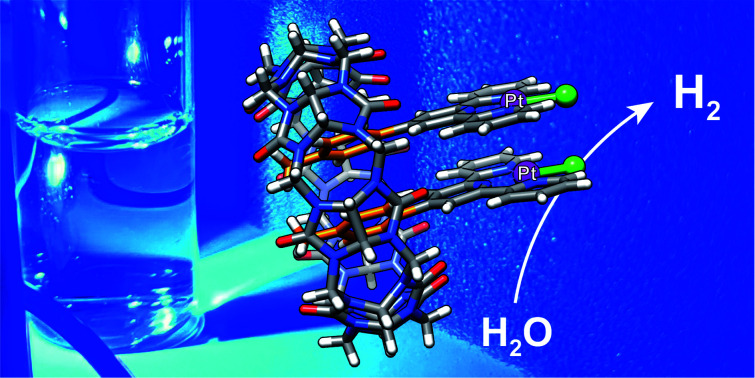

## Introduction

The design of artificial photosynthetic systems that split water into molecular hydrogen and oxygen remains a major challenge of our times.^1–16^ Explored extensively by Sakai and coworkers,^17–28^ the photoreduction of water requires a photosensitizer, an electron relay and a hydrogen-evolving catalyst, either as a three-component system,^1,2^ or with one^3–10^ or two^11,12^ multifunctional components. Sakai showed that Platinum(ii) terpyridyl (tpy) chloride 1a can be used both as a photosensitizer and H_2_-evolving catalyst in the presence of ethylenediaminetetraacetic acid (EDTA) as the sacrificial electron donor.^3^ A turnover number of approximately 3 (TON; *i.e.* the amount of H_2_ formed relative to the catalyst) was measured after 7 h in the presence of 0.4 mM of Pt complex 1a in a 2-(*N*-morpholino)ethanesulfonate (MES) buffer at pH 5.

**Fig. 1:**
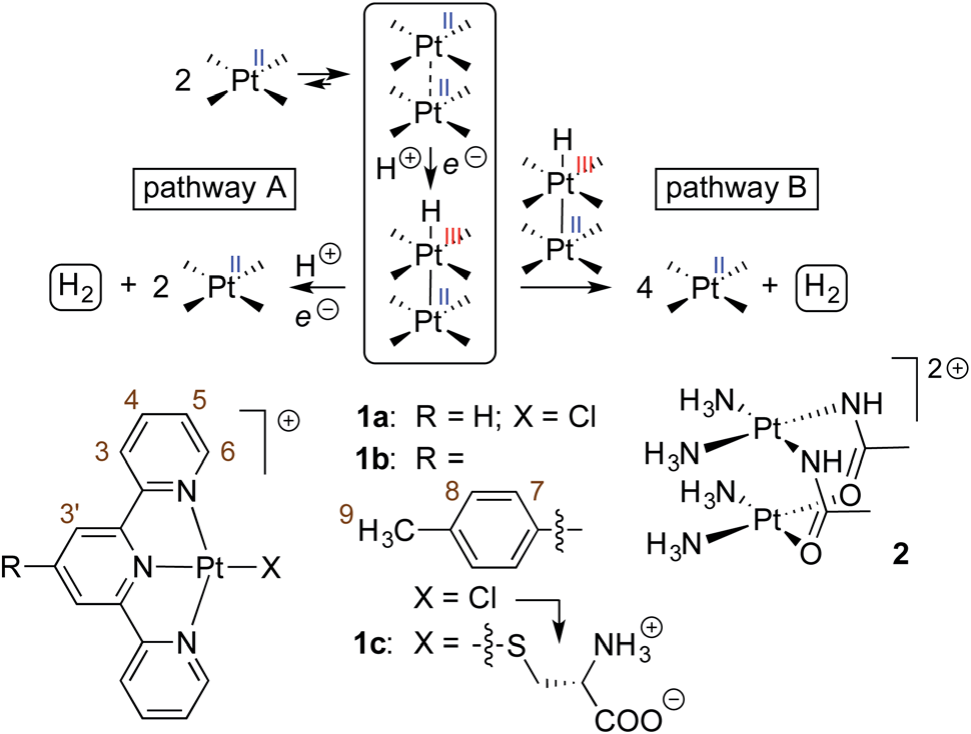
Two possible pathways for the Pt-catalyzed photoreduction of water.^13^ Pt(tpy) chloride complexes 1a^3^ and 1b and amidate-bridged dimer 2^14^ as photosensitizers and/or H_2_-evolving catalysts.

As Pt complexes are prone to dimerization caused by favorable d_*z*^2^_–d_*z*^2^_ orbital overlap of the Pt centers,^15–17^ Sakai questioned whether sensitization is triggered by complex 1a as a monomer or dimer. A dimerization constant of 4 (±2) × 10^3^ M^−1^ and the quadratic dependence of the initial rate of H_2_ production as a function of the concentration of complex 1a (below 0.4 mM) indicate that either (a) photosensitization is mostly achieved by the 1a_2_ dimer – *via* a proton-coupled electron transfer (PCET) to the corresponding Pt(ii)–Pt(iii)–H hydride (see Fig. 1, pathway A), or less likely (b) the overall rate of H_2_ formation is governed by the coupling of two Pt hydrides, a necessarily bimolecular process (see Fig. 1, pathway B).^3^

**Fig. 2:**
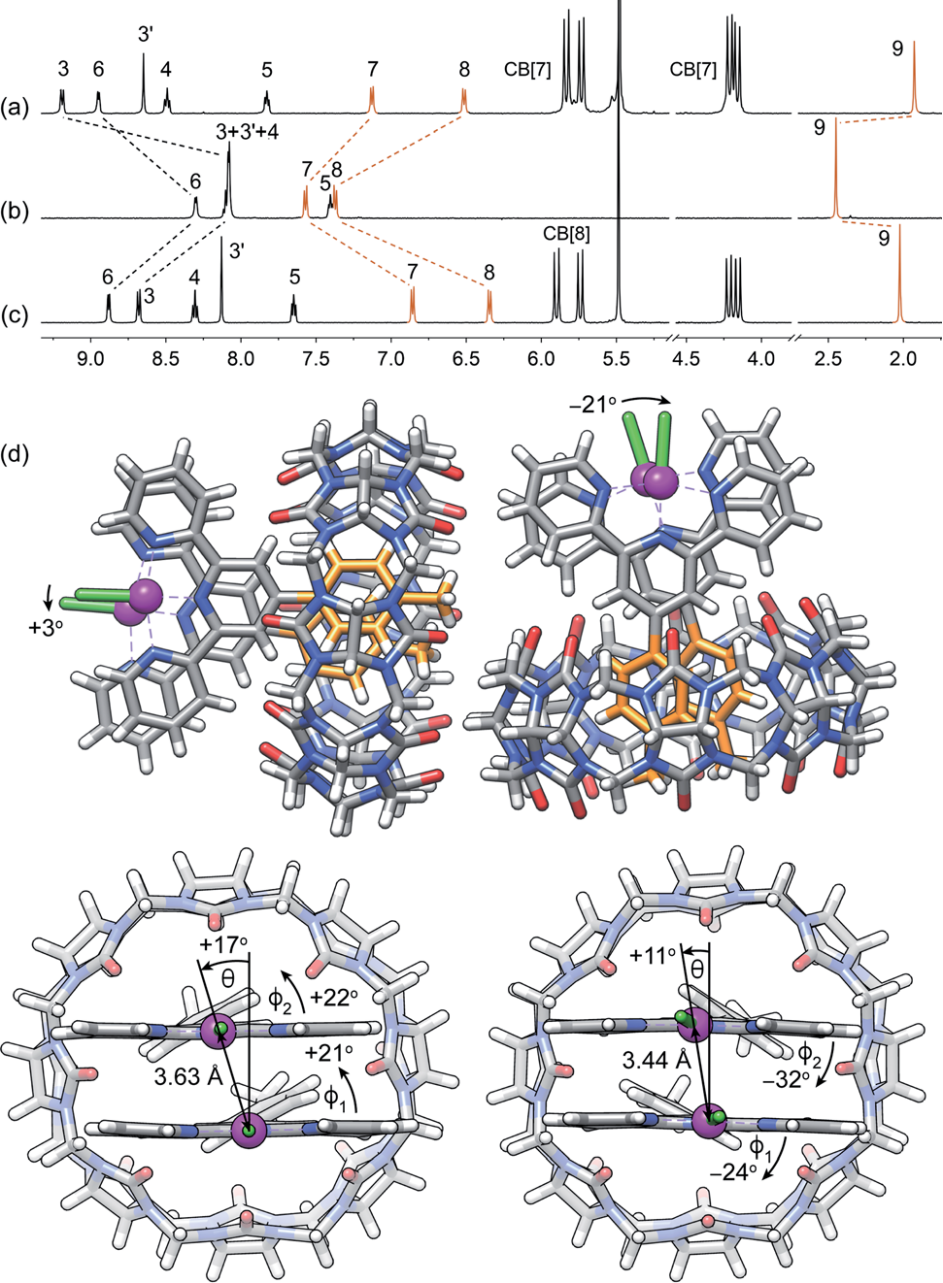
^1^H NMR spectra of complexes (a) CB[7]·1b, (b) 1b and (c) CB[8]·1b_2_ in pure D_2_O; chemical shifts in ppm; see Fig. 1 for numbering. (d) Single-crystal X-ray diffraction structure of complex CB[8]·1b_2_ showing the two conformations within the unit cell.

Sakai also showed that amidate-bridged Pt dimers (see complex 2, for example, in Fig. 1) are efficient H_2_-evolving catalysts^14,18,19^ in a three-component system consisting of the tris(bipyridine)ruthenium(ii) cation as photosensitizer and methylviologen as electron relay. Furthermore, Yamashita and coworkers showed that grafting complex 1a onto solid supports (layered niobate^20^ K_4_Nb_6_O_17_ or mesoporous silica^21,22^) improves its catalytic activity by packing the complexes into arrangements with short Pt–Pt distances. This led us to consider the cucurbit[8]uril (CB[8])-secured Pt tpy dimers prepared recently in our group as a possible new family of single component catalysts for water reduction.^23–26^ In the past few years, we have shown that Pt tpy complexes substituted at the 4′-position with CB[8]-binding aryl units form 2 : 1 head-to-head “stacked” assemblies, with both Pt tpy units sitting on top of each other at the same CB[8] portal, *i.e.* with two positively charged guests interacting with the same CB[8] portal while leaving the opposite one void of any host/guest interaction (see Fig. 2). The driving forces of this new recognition motif are dispersive interactions between the planar tpy ligands, and the very Pt–Pt interactions through d_*z*^2^_–d_*z*^2^_ orbital overlap that are suspected to enhance hydrogen formation in water reduction processes. We remind the reader that the overlap of the pair of filled d_*z*^2^_ orbitals is only favourable because of symmetry-allowed mixing with the Pt 6p_*z*_ orbitals. This returns four orbitals: a strongly bonding filled dσ, a weakly anti-bonding filled dσ* (HOMO), a weakly bonding pσ LUMO and a strongly anti-bonding pσ*. The stabilization of the dσ* HOMO confers a net energy gain to the interaction.^27,28^

## Results

Assembly CB[8]·1b_2_ was obtained *in situ* upon addition of 0.50 equiv CB[8] to solutions of Pt complex 1b in deuterium oxide, and was characterized by nuclear magnetic resonance spectroscopy (NMR). Hydrogen nuclei H^7^, H^8^ and H^9^ located at the CB[8]-binding tolyl unit experience a typical upfield shift upon encapsulation (0.71, 1.03 and 0.42 ppm, respectively; see Fig. 2, and 1 for numbering). All remaining hydrogens pertaining to the tpy ligand undergo downfield shifts caused by their proximity to the deshielding environment of the CB[8] carbonyl rim. As a control, binary assembly CB[7]·1b was prepared in a similar manner, which resulted in similar shifts (0.44, 0.85 and 0.52 ppm, respectively, for hydrogens H^7^, H^8^ and H^9^; see Fig. 2).

The X-ray crystal structure of assembly CB[8]·1b_2_ showed two different conformations within the unit cell (see Fig. 2d). This arrangement allows an optimal interaction between the carbonyl rim of one CB[8] unit with the positive Pt dimer and the outer wall of the other CB[8] macrocycle. Both CB[8]·1b_2_ conformations present slip-stacked Pt tpy units, with geometries primarily governed by the slip angle between the Pt tpy planes^29–32^ (*i.e.* the angle between the Pt–Pt axis and the normal line to one tpy NNN plane), and the C_aryl_–C_pyridyl_ torsional angles *ϕ*_1_ and *ϕ*_2_ between the CB[8]-binding tolyl group and the tpy scaffolds. In one assembly, both set of angles rotate in the same direction (*θ* = +17°, *ϕ*_1_ = +21° and *ϕ*_2_ = +22°), and in the other, the directions are opposite (*θ* = +11°, *ϕ*_1_ = −24° and *ϕ*_2_ = −32°, see Fig. 2). This results in two different Cl–Pt–Pt–Cl dihedral angles (+3° and −21°, respectively) and two Pt–Pt distances (3.63 Å and 3.44 Å, respectively). Complex 1b and its CB[*n*]-bound assemblies display typical absorption features (see Fig. S4a†). As shown by Che,^33,34^ McMillin^35–39^ and Gray,^40,41^ absorption bands between 280 and 350 nm, typically with vibronic structure and extinction coefficients of approximately 10^4^ M^−1^ cm^−1^, are associated with intra-ligand ^1^[π(tpy) → π*(tpy)] transitions (the π*(tpy) orbital being better described as a 6p_*z*_(Pt)/π*(tpy) hybrid). Absorption bands above 350 nm are broad with lower extinction coefficients; they correspond to ^1^[5d(Pt) → π*(tpy)] metal-to-ligand charge transfers (^1^MLCT).

Photolysis experiments were carried out in MES buffer (0.10 M, pH 5) in the presence of EDTA as sacrificial electron donor (30 mM). Solutions (6.0 mL in 23 mL vials equipped with PTFE septa) were irradiated with 10 royal blue LEDs (*λ* = 447 ± 10 nm; 250 mW/LED). Aliquots were removed from the headspace and analyzed by gas chromatography as a function of time (see ESI† section for details). Irradiation was intentionally carried out using the least energetic wavelengths required to trigger the photoreduction. The fact that solar irradiance at sea level peaks at approximately 460 nm (ref. 42) also guided our decision.

**Fig. 3:**
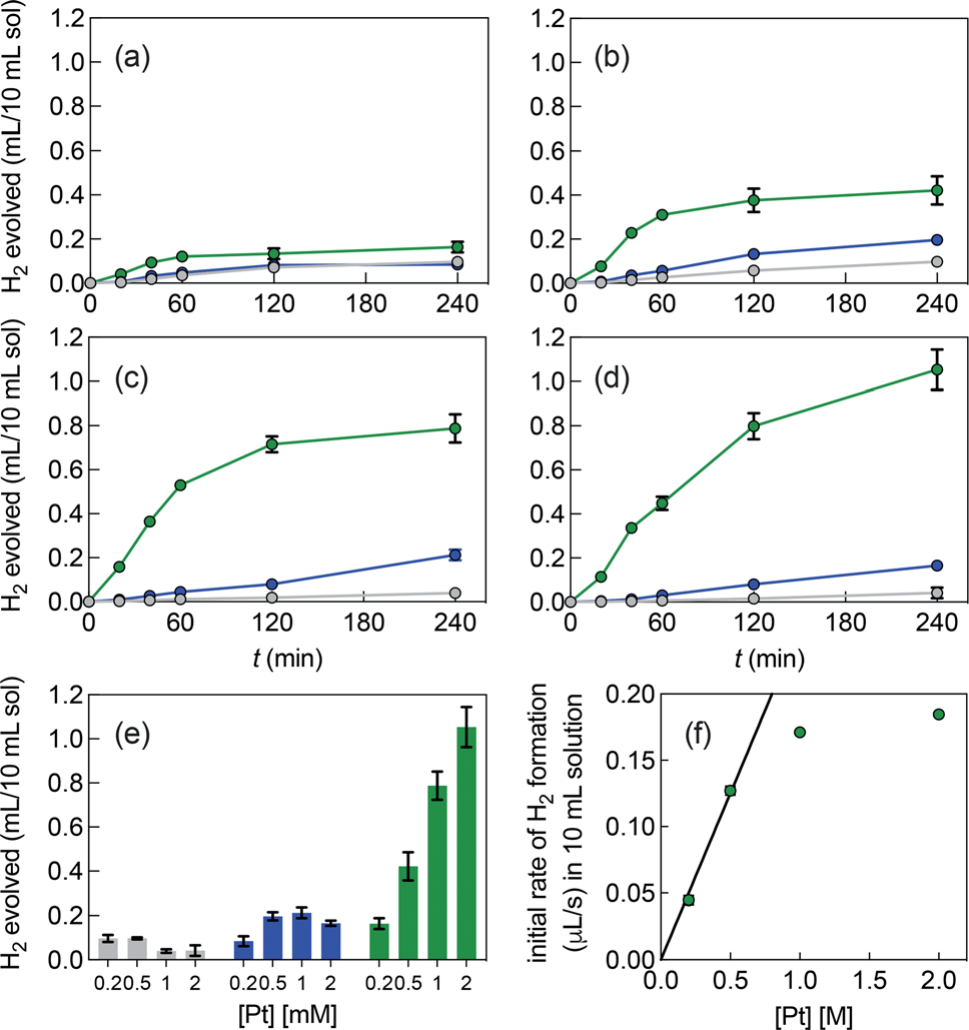
Hydrogen formation upon irradiation of aqueous solutions containing (a) 0.20 mM, (b) 0.50 mM, (c) 1.0 mM and (d) 2.0 mM of complex 1b (grey series), CB[7]·1b (blue series) and CB[8]·1b_2_ (green series). (e) H_2_ volumes formed after 4 h of irradiation under all 12 conditions. (f) Initial rates of H_2_ formation (up to 40 min) in the presence of assembly CB[8]·1b_2_ at different concentrations. All concentrations based on Pt content. 0.10 M MES buffer, pH 5, containing EDTA (30 mM). Errors are within the range covered by the data points when error bars are absent.

Fig. 3 shows the poor performance of free complex 1b at promoting water reduction (up to 0.10 mL H_2_/10 mL solution at 0.50 mM Pt after 4 h, and a TON decreasing steadily from 2.5 to 0.1 as Pt concentration increases from 0.20 to 2.0 mM). CB[7] encapsulation of the tolyl tpy substituent does not significantly enhance performance, with up to 0.20 mL H_2_/10 mL solution at 1.0 mM Pt. Increasing the catalyst load above 0.50 mM and 1.0 mM is detrimental to H_2_ production, in the case of complexes 1b and CB[7]·1b, respectively (with CB[7]·1b, The TON decreases again from 2.6 to 0.4 as Pt concentration increases from 0.20 to 2.0 mM). To the contrary, H_2_ production is increased by 25- and 6-fold when the CB[8]-secured Pt dimer is used as catalyst, compared to complexes 1b and CB[7]·1b (1.1 mL/10 mL solution at 2.0 mM Pt, compared to 0.04 and 0.17 mL, respectively). A pronounced increase in hydrogen production is observed as the catalytic load increases (see Fig. 3e and f in particular). The TON remains rather steady with Pt concentrations up to 1.0 mM (4.1, 3.7 and 3.5 at 0.20, 0.50 and 1.0 mM, respectively); a mild TON decrease to 2.4 is measured for the 2.0 mM Pt series after 4 h of irradiation. To rule out catalysis by colloidal Pt particles (if present at all), photolysis was carried out again with 1.0 mM Pt as free, CB[7] and CB[8]-bound complexes, in the presence of a few mercury drops. The latter would extract Pt(0) colloids and hamper hydrogen production if those were present and had catalytic activity.^43^ No significant changes in H_2_ formation were observed, hence ruling out this possibility. In an earlier study, Yamashita and coworkers indeed showed the absence of Pt(0) formation using X-ray absorption fine-structure spectroscopy (XFAS),^44^ after irradiation of complex 1a (4.0 mM) and EDTA (30 mM) in a sodium acetate buffer at pH 5.

Photolysis of a 0.50 mM CB[8]·1b_2_ solution was also monitored by electrospray ionization mass spectrometry, with 1.5 mM EDTA as sacrificial electron donor in water. Before irradiation, assembly CB[8]·1b_2_, its binary complex CB[8]·1b fragment, its free guest fragment, and an EDTA bridged Pt(tpy) dimer were detected (see Fig. S3†). As observed by Sakai,^3^ Pt/EDTA adducts underwent extensive decomposition upon irradiation; a noteworthy degradation product was the Pt(tpy) hydride cation (see Fig. S3 and Table S1†).

## Discussion

Fig. 3e highlights that the catalytic activity of assembly CB[7]·1b is much weaker than the corresponding CB[8]-secured Pt dimer CB[8]·1b_2_. UV-Vis experiments afford concentration-independent extinction coefficients for complex CB[7]·1b, thereby guaranteeing its presence as a monomeric species, with CB[7] preventing dimerization. As mentioned above (see Fig. 1, pathway A), photosensitization is likely enhanced when the Pt photocatalyst is present as a dimer, and complex CB[7]·1b proves to be a proper negative control in this study.

A linear correlation between the initial rate of H_2_ formation (up to 40 min) and the Pt concentration might have been detected when complex CB[8]·1b_2_ is used as the photocatalyst (see Fig. 3f). This result, in light of the possible quadratic relationship observed by Sakai with free complex 1a,^3^ could suggest that the enhanced catalytic activity of the CB[8] assembly is caused by the macrocycle securing two Pt tpy complexes together, regardless of Pt concentration. However, free Pt complexes are prone to dimerization anyway, even at low concentration, but hydrogen production in the absence of CB[8] remains low (see Fig. 3). As mentioned above, Sakai and coworkers extracted a dimerization constant of 4 (±2) × 10^3^ M^−1^ for complex 1a. UV-Vis absorption spectra of Pt complex 1b returned concentration-dependent extinction coefficients (see Fig. S4b†). The absorbances between 360 and 480 nm at various concentrations were fit with a simple dimerization model and returned a dimerization constant of 5.7 (±0.2) × 10^3^ M^−1^ in the MES/EDTA buffer, in excellent agreement with Sakai's measurements. The low solubility of free Pt complex 1b and free CB[8] in aqueous medium precluded the extraction of the thermodynamic parameters of the dimerization and CB[8] binding processes by isothermal titration calorimetry (ITC). However, the more soluble Pt thiolate 1c obtained after exchange of the chloride ligand with l-cysteine afforded a dimerization constant *K*_Pt–Pt_ of 4.0 (±0.4) × 10^4^ by ITC in water, in very good agreement with the 7-fold lower constant measured for complex 1b in the high ionic strength buffer environment. A ternary binding constant *β* for complex 1c towards CB[8] of 1.9 (±0.6) × 10^13^ M^−2^ (see equilibrium 1) was extracted. In other terms, dimer 1c_2_ (and most likely dimer 1b_2_) can be considered as a standalone guest forming a very tight 1 : 1 complex with CB[8], with a binding affinity *K*′ of 5 (±2) × 10^8^ M^−1^, obtained from eqn (2), in excellent agreement with one of our recent studies^26^ on a parent system.

1 1c + 1c + CB[8] ⇄ CB[8]·1c_2_

2
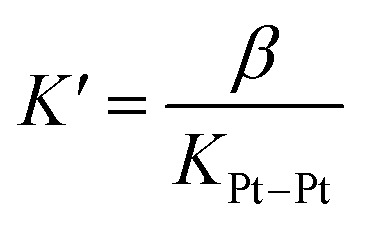


The dimerization constants of complexes 1b and 1c return free energy terms of −5.1 (±0.1) and −6.3 (±0.1) kcal mol^−1^, respectively, in excellent agreement with the typical strength of Pt–Pt interactions.^45^ They also indicate that approximately 52, 66, 74 and 81% of Pt complex 1b is already present as a dimer at concentrations of 0.2, 0.5, 1.0 and 2.0 mM, respectively, even in the absence of CB[8], in the MES/EDTA buffer. While a simple dimerization process is sufficient to fit enthalpograms and changes in UV-Vis absorption, further aggregation into trimers or larger oligomers cannot be ruled out even if, in our case, considering those processes leads to over-parametrization.

To identify the underlying cause of the catalytic enhancement in the presence of CB[8], we used density functional theory (DFT) to calculate the free energy of Pt(iii)–H hydride formation from the corresponding Pt(ii) complex (see Fig. 1), using Pt complex 1b as (1) a free monomer, (2) a CB[7]-bound monomer, (3) a dimer in the absence of CB[8], and (4) a CB[8]-secured dimer. The sheer complexity of the PCET process prevents us from calculating free energies of activation: it would necessarily involve proton transfer to the Pt center, most likely from protonated MES at pH 5, and the oxidation of a carboxylate group of EDTA to the corresponding neutral radical, both mechanisms operating with various degrees of concertedness. As these processes are unlikely to be affected by the presence of CB[*n*], we postulate that more thermodynamically favorable PCET processes would result in lower activation energies and faster hydrogen production.

Sakai notes that Pt–Pt distances obtained by DFT optimization (B3LYP/LANL2DZ)^46–49^ are often significantly longer than those measured by X-ray crystallography (0.3 Å or longer).^11^ Here, we can report that optimization in the gas phase with Grimme's newly developed B97-3c functional^50^ and def2-mTZVP basis sets^51,52^ returns accurate Pt–Pt distances and geometries highly similar to those measured by X-ray crystallography. The two CB[8]·1b_2_ arrangements (see Fig. 2d) were reoptimized at this level of theory, and afforded Pt–Pt distances of 3.71 Å (*vs.* 3.63 Å in the X-ray crystal structure) and 3.35 Å (*vs.* 3.44 Å), respectively. Cl–Pt–Pt–Cl dihedral angles are +3° and −21°, respectively, in perfect agreement with the X-ray crystal structure. Grimme also showed that for a set of reactions involving transition metals, the B97-3c functional not only returns realistic geometries, but also more accurate free energies compared to the B3LYP functional, even when the latter is supplemented with a dispersive term.^50^ The enthalpic and entropic contributions at 25 °C, as well as the free energies of solvation, were calculated using the tight-binding semi-empirical method GFN2-xTB currently developed by Grimme and coworkers^53–55^ (see ESI† section for details; GFN2-xTB reaches the accuracy of the B97-3c functional when calculating the rotational and vibrational contributions to reaction free energies, including for transition metal complexes, at a fraction of computation time).^53,54^ The HOMO in the arrangement with the short Pt–Pt distance is the expected d_*z*^2^_–d_*z*^2^_ antibonding orbital (see Fig. S6†). To the contrary, the HOMO in the other arrangement is a dxz–dxz antibonding orbital (see Fig. S7a†); d_*z*^2^_–d_*z*^2^_ antibonding is relegated to the HOMO-5 energy level (see Fig. S7b†). Remarkably, both assemblies have similar stabilities, with just a slight preference for the short Pt–Pt distance (0.4 kcal mol^−1^), thereby indicating an even competition between the Coulombic repulsion of the positively charged Pt centres and the d_*z*^2^_–d_*z*^2^_ orbital overlap. This is also consistent with the presence of both arrangements in the unit cell of the X-ray crystal structure.

Free Pt complex 1b, its CB[7]-bound assembly and dimer 1b_2_ were optimized at the same level of theory. The Cl–Pt–Pt–Cl dihedral angle in the most stable conformation of dimer 1b_2_ is 124°, and the Pt–Pt distance 4.99 Å (see Section 11 in the ESI† section). Dimerization is favoured by dispersive interactions between the planar ligands, while Pt–Pt orbital overlap is insignificant.

**Fig. 4:**
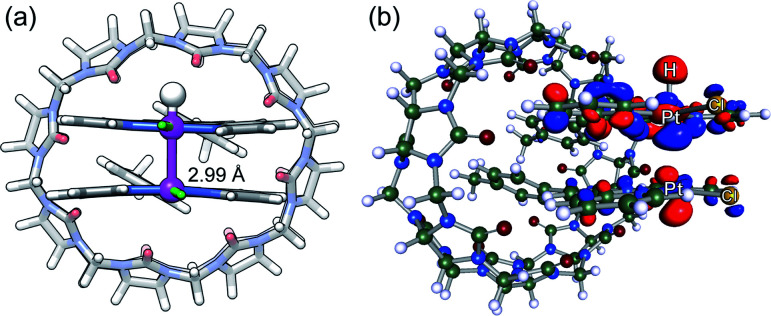
(a) DFT-optimized structure of assembly CB[8]·1b_2_–H (B97-3c/def2-mTZVP) and (b) its SOMO.

We then optimized the corresponding open-shell Pt(iii)–H hydrides at the same level of theory. As noted by Sakai, the Pt–Pt bond is reinforced compared to Pt(ii)–Pt(ii) interactions, and can be seen as a three-center two-electon Pt(ii)–Pt(iii)–H system^11,56–58^ with a Pt–Pt bond order of 0.5 (further oxidation to a d^7^–d^7^ Pt(iii)–Pt(iii) system would afford a σ bond between both metals, as the d_*z*^2^_–d_*z*^2^_ anti-bonding orbital would be empty). A unique CB[8]·1b_2_–H assembly is obtained with a pronounced shortening of the Pt–Pt bond (from 3.35 or 3.71 Å to 2.99 Å, see Fig. 4a). The 0.36 Å shortening is very consistent with that observed after oxidation of tetranuclear Pt(ii) complexes into mixed-valence Pt(ii)_2_Pt(iii)_2_ complexes.^59^ The Pt–Pt axis also crosses the tpy planes almost perpendicularly (the slip angle between both tpy planes is 2° *vs.* 11° and 17° in the pair of CB[8]·1b_2_ assemblies, see Fig. 2d). The SOMO of assembly CB[8]·1b_2_ H is indeed a d_*z*^2^_(Pt)-d_*z*^2^_(Pt)-s(H) hybrid (see Fig. 4b). A similar 2.96 Å Pt–Pt distance in dimer 1b_2_–H (with a Cl–Pt–Pt–Cl dihedral angle of 136°) was also obtained (see ESI† section).

The following observations can be made from the electronic contributions (Δ*E*) and free energies (Δ*G*) of the PCET processes (see Table 1): (1) the latter is more favourable by 4.8 kcal mol^−1^ when applied to a Pt dimer compared to a Pt monomer, even in the absence of CB[8], in strong support of the mechanism proposed by Sakai and coworkers (Δ*G* = −18.0 *vs.* −13.2 kcal mol^−1^). (2) CB[*n*] encapsulation does not provide any electrostatic contribution to the PCET process, as it is separated from the Pt centers by the tpy ligands (Δ*G* = −13.6 *vs.* −13.2 kcal mol^−1^ in the presence and absence of CB[7], respectively). (3) Aggregation of free Pt complex 1b must severely hamper the catalytic process, as calculations indicate a more favorable PCET process for dimer 1b_2_ compared to complex CB[7]·1b (Δ*G* = −18.0 *vs.* −13.6 kcal mol^−1^), whereas hydrogen production is even poorer. (4) Most importantly, in addition to efficiently deaggregating Pt complex 1b (and likely preventing the formation of higher order oligomers), CB[8] secures the Pt dimer into a conformation that facilitates the PCET process. If dimerization of Pt complex 1b were quantitative in the presence and absence of CB[8], a 2.2 kcal mol^−1^ gain for the PCET process would be obtained upon addition of the macrocycle (Δ*G* = −20.2 *vs.* −18.0 kcal mol^−1^ for dimer 1b_2_, in the presence and absence of CB[8], respectively, see Table 1). However, dimerization is only partial in the absence of CB[8]; to allow a proper comparison, the calculated free energy Δ*G* of the PCET process must be corrected by −*RT* ln *x*, where *x* is the fraction of Pt complex 1b present as a dimer (74% at 1.0 mM, for example, *i.e.* a correction of −0.17 kcal mol^−1^). The energy gain upon addition of CB[8] therefore ranges from 1.8 to 2.1 kcal mol^−1^ depending on Pt concentration. Also, removing CB[8] from complexes CB[8]·1b_2_ and CB[8]·1b_2_–H and carrying out single point energy calculations returns an electronic contribution Δ*E* of −31.4 kcal mol^−1^ for the PCET process, a 4.6 kcal mol^−1^ improvement compared to dimer 1b_2_ in its most stable conformation in the absence of CB[8] (Δ*E* = −26.8 kcal mol^−1^). In addition to being computed using adequate functionals and semi-empirical methods, we note that the quality of these calculated energy gains must be further enhanced by error compensation, as they represent relative stabilities of very similar assemblies. One can thus conclude that CB[8] encapsulation destabilizes dimer 1b_2_ by at least 1.8 kcal mol^−1^, enhances its reactivity, and promotes hydrogen production.

**Table 1:** Coulombic (Δ*E*) and free energy (Δ*G*) terms for Pt(iii)–H hydride formation from Pt(ii) complexes *via* PCET processes

PCET process	Δ*E*^a^	Δ*G*^a^
1b^+^ + H → [1b–H]^+^	−21.8	−13.2
CB[7]·1b^+^ + H → [CB[7]·1b–H]^+^	−20.7	−13.6
1b_2_^2+^ + H → [1b_2_–H]^2+^	−26.8	−18.0
CB[8]·1b_2_^2+^ + H → [CB[8]·1b_2_–H]^2+^	−28.7	−20.2

a In kcal mol^−1^.

## Conclusions

While noble metal-free catalysts and chromophores can promote water reduction efficiently,^60–62^ we showed here, as a proof of concept with arguably modest TONs, that a macrocycle like CB[8] can be used as a tool to enhance the catalytic activity of a well-established family of multifunctional photocatalysts, namely Pt complexes. CB[8] effectively secures a pair of Pt complexes 1b on top of one another into reactive dimer CB[8]·1b_2_, which upon irradiation yields volumes of hydrogen up to 25 and 6 times larger than those obtained with the corresponding free monomer 1b and the CB[7]-bound control CB[7]·1b, respectively. A combination of semi-empirical and DFT calculations suggest that the kinetics and activity of the photocatalysts are governed by the thermodynamics of the PCET reaction from the Pt(ii) complexes to the corresponding Pt(iii)–H hydride intermediates. CB[8]-promoted dimerization not only renders the process more exergonic (by 4.8 kcal mol^−1^), as the PCET process is more favourable starting from a Pt(ii) dimer compared to a monomer, but also by forcing the dimer into a more reactive conformation for Pt(ii)–Pt(iii)–H hydride formation (an approximately 2 kcal mol^−1^ free energy gain).

## Data availability

All analytical data is provided in the ESI.† All details about computational methods and associated coordinates are available in the ESI.†

## Author contributions

EM conceived the project, and mentored RR, MN and HB; TAW mentored SS; TAW and SS designed the photolysis experiments. NK determined the single-crystal X-ray diffraction structure of assembly CB[8]·1b_2_. RR, SS, HB and MN conducted all other experiments. EM, RR, SS, HB and MN wrote the manuscript.

## Conflicts of interest

There are no conflicts to declare.

## Supplementary Material

SC-012-D1SC03743A-s001

SC-012-D1SC03743A-s002

## References

[cit1] Yamauchi K., Masaoka S., Sakai K. (2009). J. Am. Chem. Soc..

[cit2] Tanaka S., Nakazono T., Yamauchi K., Sakai K. (2017). Chem. Lett..

[cit3] Okazaki R., Masaoka S., Sakai K. (2009). Dalton Trans..

[cit4] Masaoka S., Mukawa Y., Sakai K. (2010). Dalton Trans..

[cit5] Kobayashi M., Masaoka S., Sakai K. (2012). Dalton Trans..

[cit6] Yamamoto K., Kitamoto K., Yamauchi K., Sakai K. (2015). Chem. Commun..

[cit7] Yamauchi K., Sakai K. (2015). Dalton Trans..

[cit8] Kitamoto K., Sakai K. (2016). Chem. Commun..

[cit9] Lin S., Kitamoto K., Ozawa H., Sakai K. (2016). Dalton Trans..

[cit10] Yatsuzuka K., Yamauchi K., Sakai K. (2021). Chem. Commun..

[cit11] Ogawa M., Ajayakumar G., Masaoka S., Kraatz H. B., Sakai K. (2011). Chem.–Eur. J..

[cit12] Kobayashi M., Masaoka S., Sakai K. (2012). Angew. Chem., Int. Ed..

[cit13] Ozawa H., Sakai K. (2011). Chem. Commun..

[cit14] Sakai K., Ozawa H. (2007). Coord. Chem. Rev..

[cit15] Williams J. A. G. (2007). Top. Curr. Chem..

[cit16] Eryazici I., Moorefield C. N., Newkome G. R. (2008). Chem. Rev..

[cit17] Mauro M., Aliprandi A., Septiadi D., Kehr N. S., De Cola L. (2014). Chem. Soc. Rev..

[cit18] Sakai K., Matsumoto K. (1988). J. Coord. Chem..

[cit19] Sakai K., Matsumoto K. (1990). J. Mol. Catal..

[cit20] Mori K., Ogawa S., Martis M., Yamashita H. (2012). J. Phys. Chem. C.

[cit21] Mori K., Watanabe K., Fuku K., Yamashita H. (2012). Chem.–Eur. J..

[cit22] Mori K., Watanabe K., Terai Y., Fujiwara Y., Yamashita H. (2012). Chem.–Eur. J..

[cit23] Kotturi K., Masson E. (2018). Chem.–Eur. J..

[cit24] Thompson N. A., Barbero H., Masson E. (2019). Chem. Commun..

[cit25] Barbero H., Thompson N. A., Masson E. (2020). J. Am. Chem. Soc..

[cit26] Barbero H., Masson E. (2021). Chem. Sci..

[cit27] John E. B., Alec C. D., Gray H. B., Green J. C., Hazari N., Labinger J. A., Jay R. W. (2010). Inorg. Chem..

[cit28] Darnton T. V., Hunter B. M., Hill M. G., Záliš S., Vlček A., Gray H. B. (2016). J. Am. Chem. Soc..

[cit29] Kasha M., Rawls H. R., El-Bayoumi M. A. (1965). Pure Appl. Chem..

[cit30] Hartnett P. E., Timalsina A., Matte H. S. S. R., Zhou N., Guo X., Zhao W., Facchetti A., Chang R. P. H., Hersam M. C., Wasielewski M. R., Marks T. J. (2014). J. Am. Chem. Soc..

[cit31] Margulies E. A., Shoer L. E., Eaton S. W., Wasielewski M. R. (2014). Phys. Chem. Chem. Phys..

[cit32] Gemen J., Ahrens J., Shimon L. J. W., Klajn R. (2020). J. Am. Chem. Soc..

[cit33] Yip H., Cheng L., Cheung K., Che C. (1993). J. Chem. Soc., Dalton Trans..

[cit34] Clo C., Me C. H. C. O., Lai S., Chan M. C. W., Cheung K., Che C. (1999). Inorg. Chem..

[cit35] Aldridge T. K., Stacy E. M., McMillin D. R. (1994). Inorg. Chem..

[cit36] Büchner R., Field J. S., Haines R. J., Cunningham C. T., McMillin D. R. (1997). Inorg. Chem..

[cit37] Crites D. K., Cunningham C. T., McMillin D. R. (1998). Inorg. Chim. Acta.

[cit38] Michalec J. F., Bejune S. A., Cuttell D. G., Summerton G. C., Gertenbach J. A., Field J. S., Haines R. J., McMillin D. R. (2001). Inorg. Chem..

[cit39] Büchner R., Cunningham C. T., Field J. S., Haines R. J., McMillin D. R., Summerton G. C. (1999). J. Chem. Soc., Dalton Trans..

[cit40] Bailey J. A., Hill M. G., Marsh R. E., Miskowski V. M., Schaefer W. P., Gray H. B. (1995). Inorg. Chem..

[cit41] Hill M. G., Bailey J. A., Miskowski V. M., Gray H. B. (1996). Inorg. Chem..

[cit42] Gates D. M. (1966). Science.

[cit43] Du P., Schneider J., Li F., Zhao W., Patel U., Castellano F. N., Eisenberg R. (2008). J. Am. Chem. Soc..

[cit44] Martis M., Mori K., Kato K., Sankar G., Yamashita H. (2013). ChemPhysChem.

[cit45] Jennette K. W., Gill J. T., Sadownick J. A., Lippard S. J. (1976). J. Am. Chem. Soc..

[cit46] Hay P. J., Wadt W. R. (1985). J. Chem. Phys..

[cit47] Wadt W. R., Hay P. J. (1985). J. Chem. Phys..

[cit48] Hay P. J., Wadt W. R. (1985). J. Chem. Phys..

[cit49] Becke A. D. (1993). J. Chem. Phys..

[cit50] Brandenburg J. G., Bannwarth C., Hansen A., Grimme S. (2018). J. Chem. Phys..

[cit51] Weigend F., Ahlrichs R. (2005). Phys. Chem. Chem. Phys..

[cit52] Weigend F. (2006). Phys. Chem. Chem. Phys..

[cit53] Grimme S., Bannwarth C., Shushkov P. (2017). J. Chem. Theory Comput..

[cit54] Bannwarth C., Ehlert S., Grimme S. (2019). J. Chem. Theory Comput..

[cit55] Grimme S. (2019). J. Chem. Theory Comput..

[cit56] O'halloran T. V., Mascharak P. K., Williams I. D., Roberts M. M., Lippard S. J. (1987). Inorg. Chem..

[cit57] Matsumoto K., Sakai K. (1999). Adv. Inorg. Chem..

[cit58] Lippert B. (1999). Coord. Chem. Rev..

[cit59] Sakai K., Tanaka Y., Tsuchiya Y., Hirata K., Tsubomura T., Iijima S., Bhattacharjee A. (1998). J. Am. Chem. Soc..

[cit60] Zou X., Zhang Y. (2015). Chem. Soc. Rev..

[cit61] Yuan Y.-J., Yu Z.-T., Chen D.-Q., Zou Z.-G. (2017). Chem. Soc. Rev..

[cit62] Lu H., Tournet J., Dastafkan K., Liu Y., Ng Y. H., Karuturi S. K., Zhao C., Yin Z. (2021). Chem. Rev..

