# Supplementary material for: Enhanced photoreduction of water catalyzed by a cucurbit[8]uril-secured platinum dimer
Source: Chem Sci. 2021 Nov 4;12(46):15347–52. doi: 10.1039/d1sc03743a (PMC8635170; doi:10.1039/d1sc03743a)
Supplement: SC-012-D1SC03743A-s001 [file SC-012-D1SC03743A-s001.pdf]

Electronic Supplementary Material (ESI) for Chemical Science.
This journal is © The Royal Society of Chemistry 2021

Enhanced photoreduction of water catalyzed by a cucurbit[8]uril-secured platinum dimer

Ramin Rabbani,[a†] Sima Saeedi,[a†] Md Nazimuddin,[a] Héctor Barbero,[a,b] Nathalie Kyritsakas,[c] Travis A. White,[a] and Eric Masson*[a]

[a] Department of Chemistry and Biochemistry, Ohio University, Athens, Ohio 45701, United States

[b] GIR MIOMeT, IU CINQUIMA/Química Inorgánica, Facultad de Ciencias, Universidad de Valladolid, E47011, Valladolid, Spain.

[c] Molecular Tectonics Laboratory, University of Strasbourg, UMR UDS-CNRS 7140, Institut le Bel, F-67000 Strasbourg, France.

[†] These authors contributed equally.

e-mail: masson@ohio.edu

Supporting Information

1. Generalities 2
2. Preparation and characterization of CB[n]-bound Pt(tpy) chloride complexes 2
3. Photocatalytic H_2 production 4
4. Chemical actinometry 4
5. Mass spectrometry analysis during the photolysis of complex CB[8]·**1b**$_2$ 5
6. UV-Vis characterization 8
7. Isothermal titration calorimetry 8
8. X-ray crystallography 9
9. Computational details 12
10. Coordinates of optimized structures 12
11. Frontier orbitals selection for assembly CB[8]·**1b**$_2$ 18
12. References 19

1. Generalities

All reagents were purchased from chemical suppliers and used without further purification. Cucurbit[7]uril (CB[7]) and Cucurbit[8]uril (CB[8]) were prepared using known procedures.[1] Solvents were of analytical grade and either used as purchased or dried according to procedures described elsewhere.[2] Characterization by nuclear magnetic resonance spectroscopy (NMR) was carried out using a Bruker Ascend 500 MHz spectrometer. ^{1}H and ^{13}C NMR chemical shifts are reported in parts per million (ppm) and are referenced to TMS using the residual signal of the solvent as an internal reference. Coupling constants (J) are reported in hertz (Hz). Standard abbreviations used to indicate multiplicity are: s = singlet, d = doublet, dd = doublet of doublets, t = triplet. High resolution electrospray ionization mass spectrometry (HR-ESI-MS) was performed using a Thermo Fisher Scientific Q Exactive Plus hybrid quadrupole−Orbitrap mass spectrometer in positive mode. UV-Vis absorption spectra were recorded on an Agilent HP-8453 diode-array spectrophotometer. Wavelengths (λ) are reported in nanometers (nm) and molar absorption coefficients (ε) are reported in $M^{-1}cm^{-1}$. Computational work was carried out on the Owens cluster of the Ohio Supercomputer Center in Columbus, OH (23,392-core Dell Intel Xeon E5-2680 v4 machines). 4'-(p-tolyl)-2,2':6',2"-terpyridine and chloro[4'-(p-tolyl)-2,2':6',2"-terpyridine]-platinum(II) chloride (**1b**) were prepared according to published procedures.[3]

2. Preparation and characterization of CB[n]-bound Pt(tpy) chloride complexes

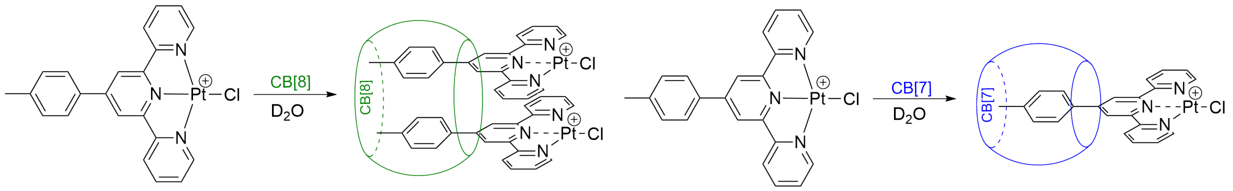

1b CB[8]·**1b**$_2$ **1b** CB[7]·**1b**

Chloro[4'-(p-tolyl-2,2':6',2"-terpyridine]platinum(II) chloride (**1b**) (3.1 mg, 5.1 μmol) was mixed with D_2O (5.0 mL). CB[8] (3.3 mg, 2.5 μmol) or CB[7] (5.9 mg, 5.1 μmol) was added subsequently, and the resulting mixture was sonicated thoroughly. The stock solution was stored at 4 ºC for further use.

Spectral data of assembly CB[8]·**1b**$_2$ have been previously reported by us elsewhere.[4]

CB[7]·**1b**. ^{1}H NMR (500 MHz, D_2O) δ 9.19 (d, J = 8.1 Hz, 2H, H^{3}), 8.95 (d, J = 5.7 Hz, 2H, H^{6}), 8.65 (s, 2H, $H^{3'}$), 8.49 (t, J = 7.8 Hz, 2H, H^{4}), 7.83 (t, J = 6.7 Hz, 2H, H^{5}), 7.13 (d, J = 7.8 Hz, 2H, H^{7}), 6.51 (d, J = 8.0 Hz, 2H, H^{8}), 5.83 (d, J = 15.4 Hz, 7H, $H^{CB[7]}$), 5.73 (d, J = 15.4 Hz, 7H, $H^{CB[7]}$), 5.48 (s, 14H, $H^{CB[7]}$), 4.19 (dd, J = 25.4, 15.4 Hz, 14H, $H^{CB[7]}$), 1.93 (s, 3H, H^{9}). ^{13}C NMR (126 MHz, D_2O) δ 159.59, 156.13, 156.08, 154.95, 153.60, 150.90, 142.48, 142.13, 132.34, 128.42, 127.74, 126.63, 126.20, 123.24, 71.22, 52.52, 20.50. HRMS (ESI): m/z = 869.205161 $[M+Na]^{2+}$ (calcd. 869.204037 for $C_{64}H_{59}ClN_{31}O_{14}PtNa$).

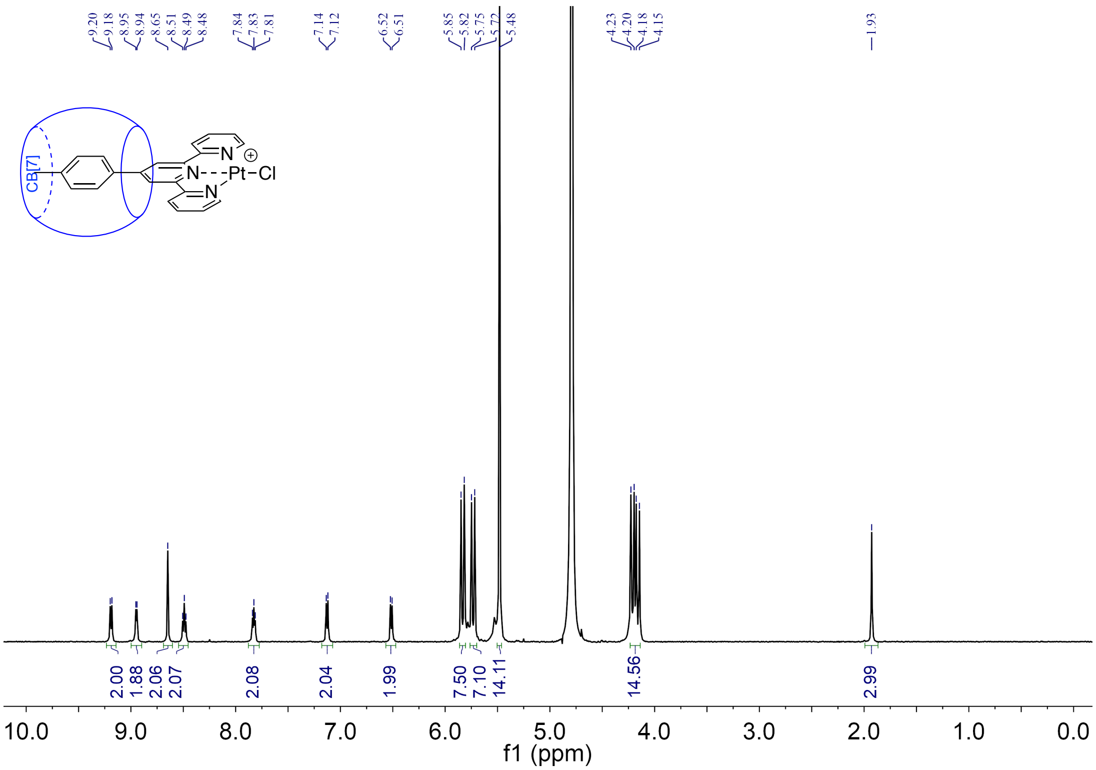

Figure S1. ^{1}H-NMR spectrum of complex CB[7]·**1b** in D_2O.

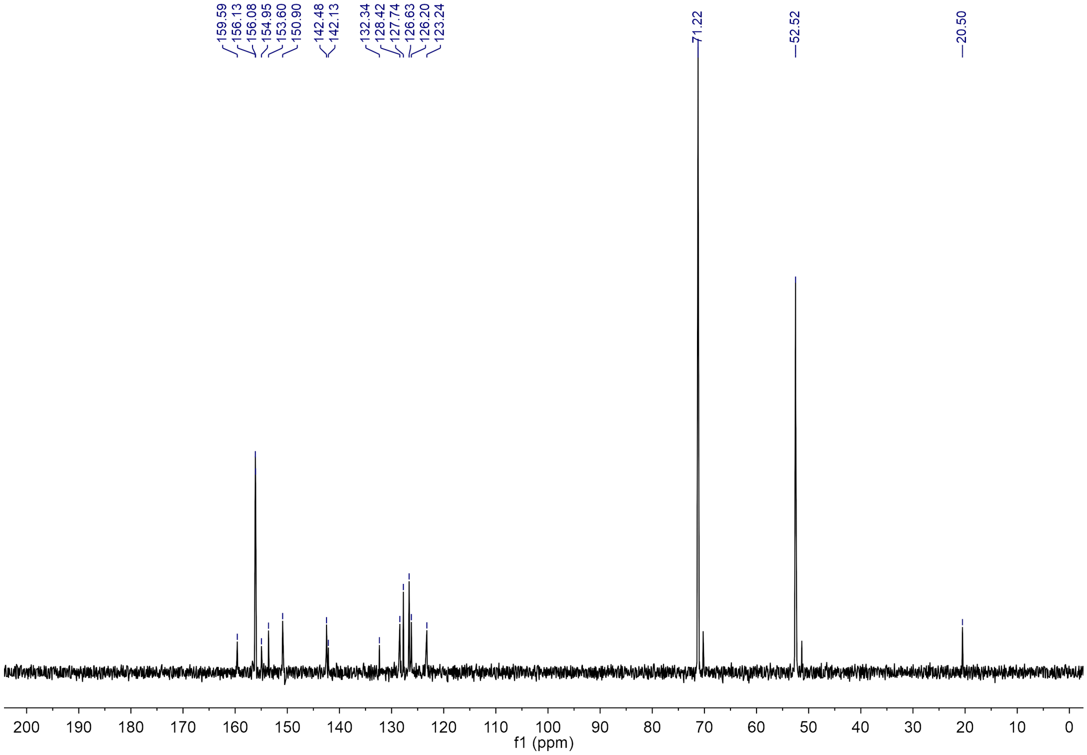

Figure S2. ^{13}C{^{1}H}-NMR spectrum of complex CB[7]·**1b** in D_2O.

3. Photocatalytic H_2 production

All photolysis experiment were carried out under buffered condition (pH 5, 0.10 M MES, 30 mM EDTA). Photolysis experiments were carried out at least in duplicates for each reaction condition. Each replicate had 6.0 mL of the solution in 23.0 mL vials equipped with PTFE/silicone septa caps. Samples were prepared in the dark and deoxygenated for 10 min using Ar prior to photolysis. Each solution was photolyzed using in-house built royal blue LEDs (λ^{irr} = 447.5 ± 10 nm at fwhm) purchased from Luxeon Star LEDs (Quadica Developments, Inc., Lethbridge, Alberta, Canada). The output from each LED was 250 mW, and samples were placed 1 cm away from the light source. After photolyzing each solution, an aliquot (0.10 mL) was removed from the headspace using a Hamilton GASTIGHT syringe and injected into a Shimadzu GC-2014 gas chromatograph (GC; Ar carrier gas) with a packed ShinCarbon ST SilicoSmooth stainless steel column (2 m long × 1/8 in.o.d. × 2.0 mm i.d.; 80/100 mesh) and a Shimadzu TCD-2014 thermal conductivity detector. The GC conditions were as follows: injector temperature, 41 °C; column temperature, 30 °C; detector temperature, 150 °C; gas flow, 25 mL/min. The volume of injected H_2 was determined using a calibration curve generated from known volumes of 100% H_2.

4. Chemical actinometry

Chemical actinometry was carried out according to literature sources.[5,6] All sample preparations and photolysis experiments were performed in the dark and in triplicates. $K_3[Fe(C_2O_4)_3]$ (6.0 mL, 0.15 M) in a 23 mL vial was photolyzed for 5 s with 447.5 ± 10 nm LED irradiation. An aliquot of the photolyzed solution (10 mL) was added to 0.1% buffered 1,10-phenanthroline (phen) solution (5.0 mL). The mixture was kept in the dark for 1 h. The process was repeated with irradiation times of 10, 15 and 30 s; a control experiment without irradiation was also carried out. Solutions were transferred into 10 mm cuvettes and their absorbances were measured using an Agilent Cary 8454 diode array UV−vis spectrophotometer (1 nm resolution, 0.5 s integration time).

Amounts of Fe^{2+} ions $n(Fe^{2+})$ produced during photolysis were obtained as follows:

$$n(\mathrm{Fe}^{2+}) = \frac{V_1\, V_3\, \Delta A_{510}}{\varepsilon_{510}\, l\, V_2} \qquad (1)$$

where ΔA_{510} is the absorbance difference at 510 nm, l the path length (10 mm), ε_{510} the extinction coefficient of $[Fe(phen)_3]^{2+}$ at 510 nm (11,100 $M^{-1}\cdot cm^{-1}$), V_1 the total volume of irradiated solution (6.0 mL), V_2 the volume of the aliquot taken from the irradiated solution (10 mL) and V_3 the volume that the aliquot is diluted into (5.0 mL).

The photon flux ($q_{n,p}$) was calculated as follows:

$$q_{n,p} = \frac{moles\ Fe^{2+}}{\Phi(\lambda)\ t} \qquad (2)$$

where $\Phi(\lambda)$ is the reported quantum yield of $K_3[Fe(C_2O_4)_3]$ photodegradation at wavelength λ and t the irradiation time (s).

5. Mass spectrometry analysis during the photolysis of complex CB[8]·1b$_2$

Photolysis prior to HR-ESI-MS analysis was carried out on a 0.50 mM solution of assembly CB[8]·**1b**$_2$ in a 1.5 mM EDTA solution in LC-MS grade water. An aliquot (10 µL) was withdrawn from the solution at specific times, diluted 10 times with a 1:1 solution of water (LC-MS grade) and methanol (HPLC grade), and injected into the mass spectrometer.

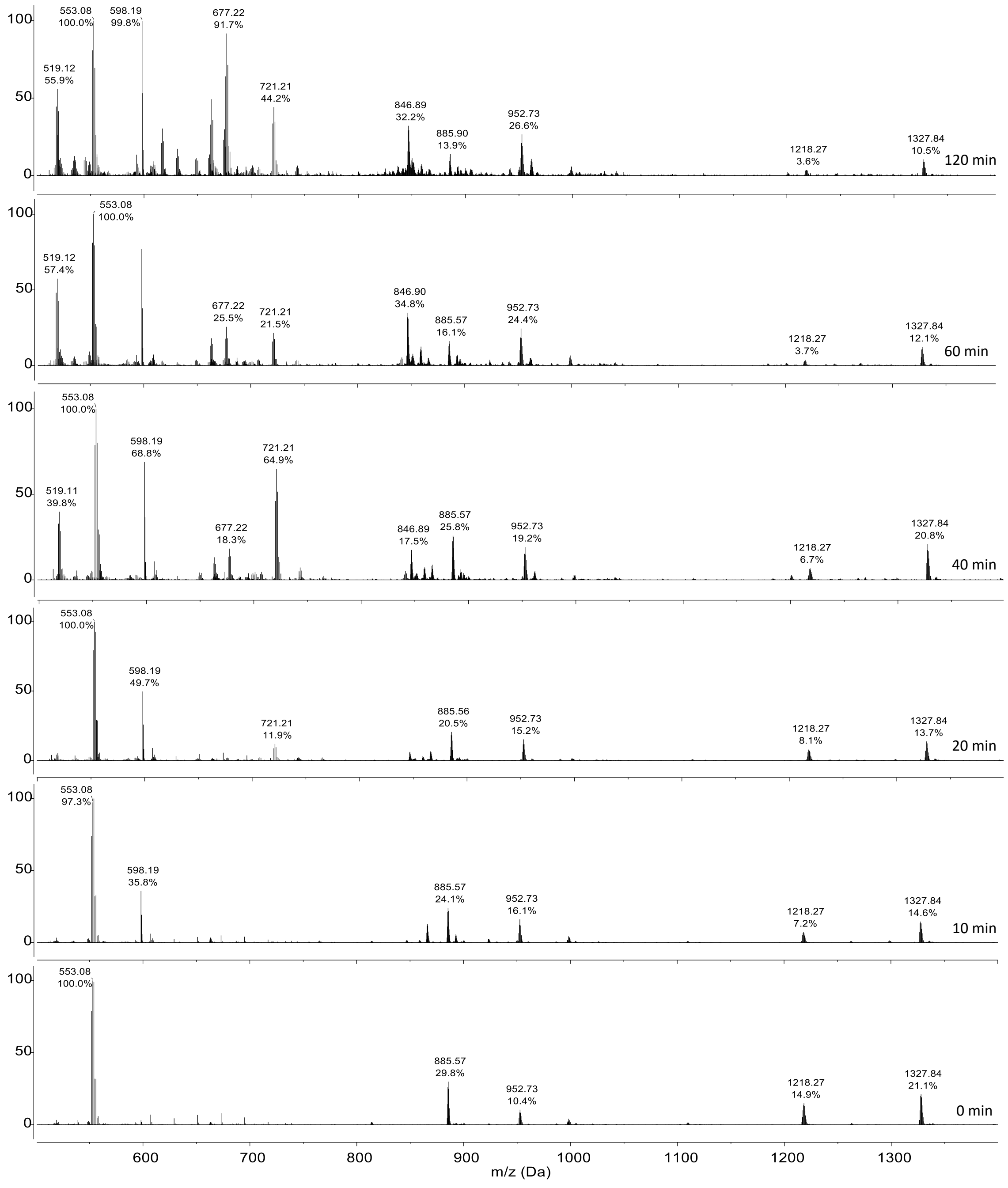

Figure S3. Bottom to top: ESI-MS analysis of photolyzed solutions of complex CB[8]·**1b**$_2$ (0.50 mM; EDTA 1.5 mM) after 0, 10, 20, 30, 40, 60 and 120 min irradiation at 447 nm.

Table S1. ESI-MS analysis of photolyzed solutions of complex CB[8]·**1b**$_2$ (0.50 mM Pt; EDTA 1.5 mM); observed (top) and calculated (bottom) positive-ion spectra.

m/z 519.12	.
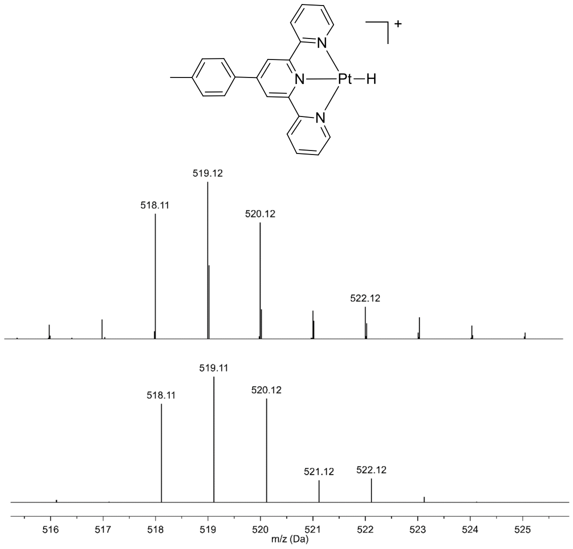	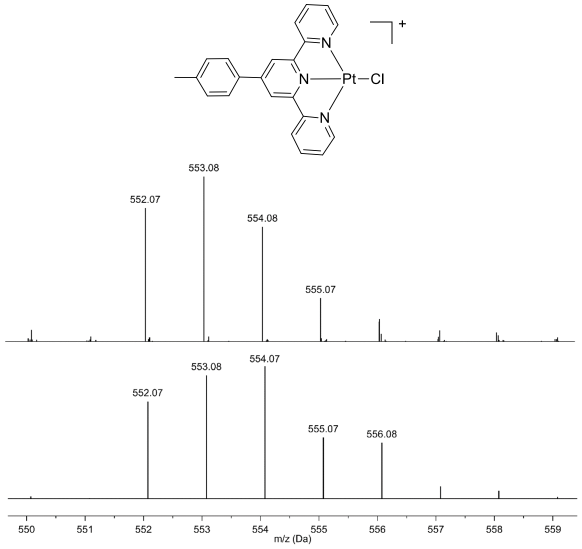

m/z 598.19	*m/z* 677.22
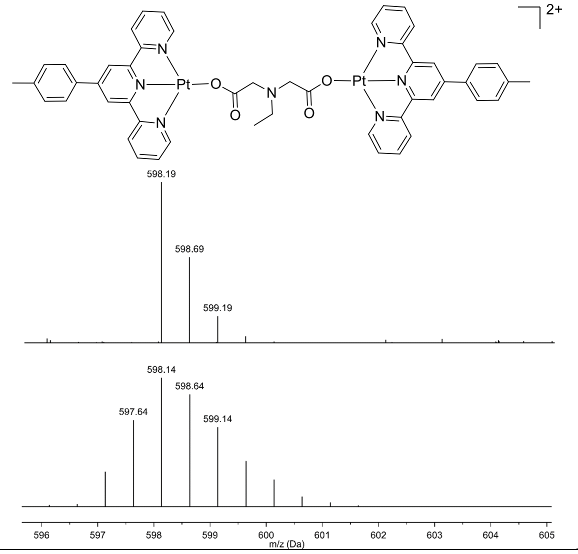	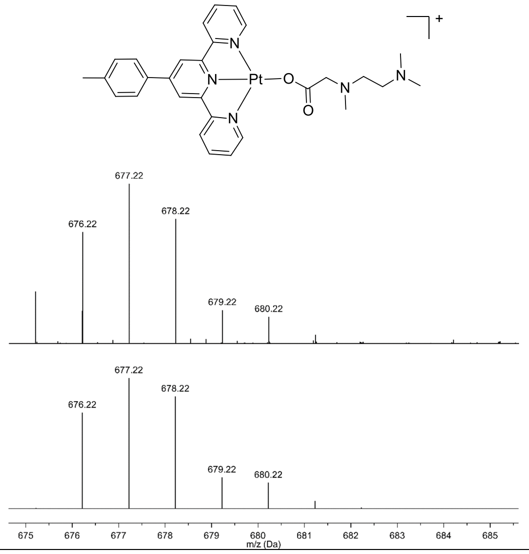

m/z 721.21	*m/z* 885.90

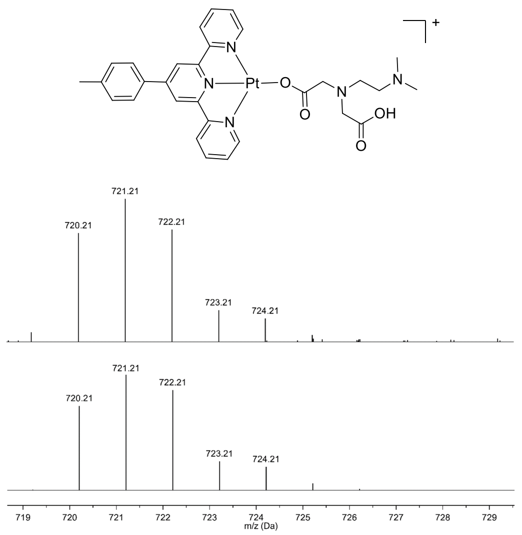

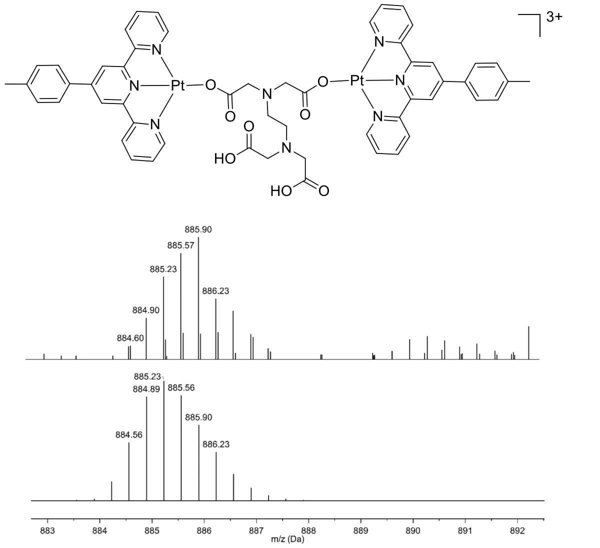

m/z 952.73	*m/z* 1218.27

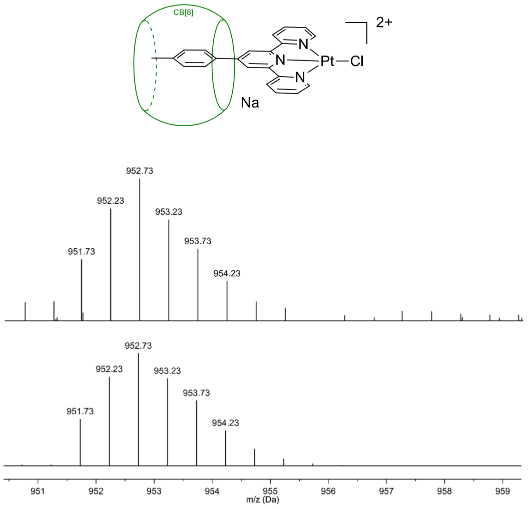

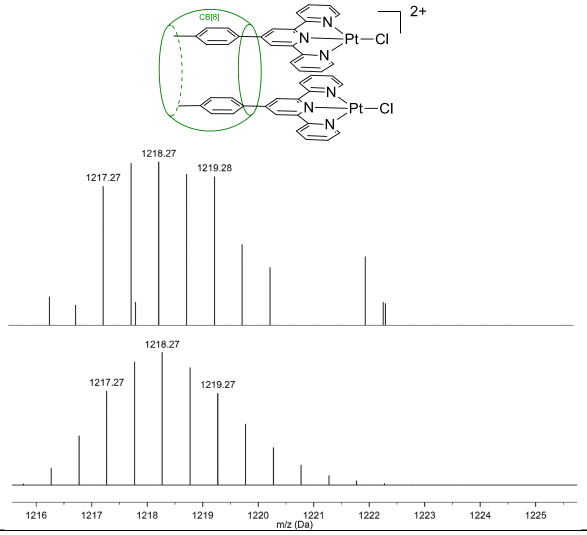

m/z 1327.84
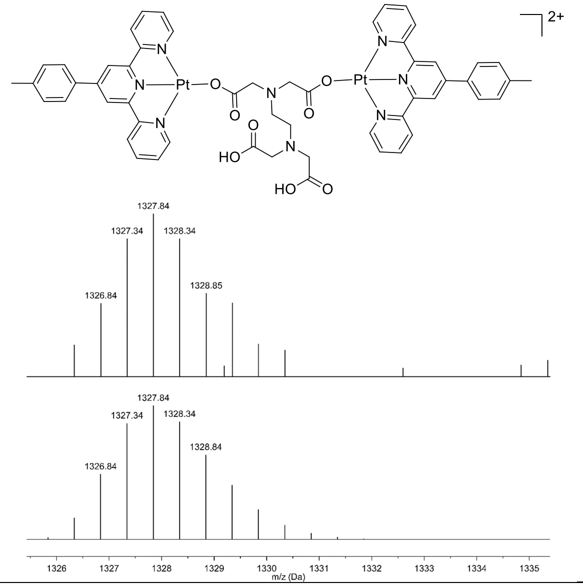

6. UV-Vis characterization

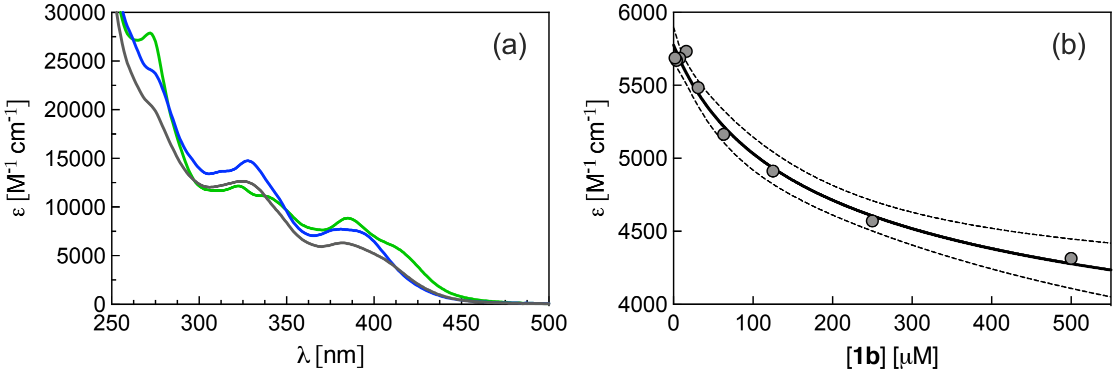

Figure S4. (a) UV-Vis absorption spectra of complex **1b** (in grey), binary assembly CB[7]·**1b** (in blue) and ternary assembly CB[8]·**1b**$_2$ (in green); Pt concentration: 20 μM. (b) Extinction coefficients of complex **1b** solutions at 384 nm as a function of concentration; fit with a dimerization model (solid black line) and 95% confidence interval (dashed lines). All spectra recorded in MES (0.10 M)/EDTA (30 mM) buffer.

7. Isothermal titration calorimetry

All isothermal titration calorimetry (ITC) experiments were carried out in MilliQ water at 25 ºC on a Malvern MicroCal ITC200 instrument. Due to the low solubility of complex **1b** and CB[8], L-Cys-derived surrogate **1c** was used as the titrant instead. The latter was set up in the injection syringe at concentrations ranging from 0.85 to 1.0 mM. Hosts (CB[7] and CB[8]) were in the sample cell at concentrations ranging from 0.050 to 0.10 mM. Experiments were carried out in triplicate. Each titration consisted of 20 injections with an injection spacing of 150 s. Raw data were analyzed (baseline correction, integration and fitting) with Affinimeter software.

Table S2. Thermodynamic data obtained by ITC titrations of the species involved in this study.

System	K	ΔG [c]	ΔH [c]	$T\Delta S$ [c]	$K_{Pt\text{-}Pt}$ [d]	$\Delta G_{Pt\text{-}Pt}$ [c]	$\Delta H_{Pt\text{-}Pt}$ [c]	$T\Delta S_{Pt\text{-}Pt}$ [c]
**1c vs CB[7]** [a]	6.3 (±0.1) × 10^4	–6.55 (±0.01)	–5.87 (±0.04)	0.69 (±0.04)	-	-	-	-
**1c vs CB[8]**	1.9 (±0.6) × 10^{13} [b]	–18.1 (±0.2) [b]	–19.4 (±0.2) [b]	–1.3 (±0.3) [b]	4.0 (±0.4) × 10^4	–6.27 (±0.06)	-2.8 (±0.3)	3.5 (±0.3)

[a] Binding constant in M^{-1}. [b] Thermodynamic parameter corresponding to the equilibrium $\mathbf{1c} + \mathbf{1c} +$ CB[8] ⇄ CB[8] · $\mathbf{1c}_2$, in M^{-2}. [c] in kcal/mol. [d] Dimerization constant of complex **1c** [M^{-1}].

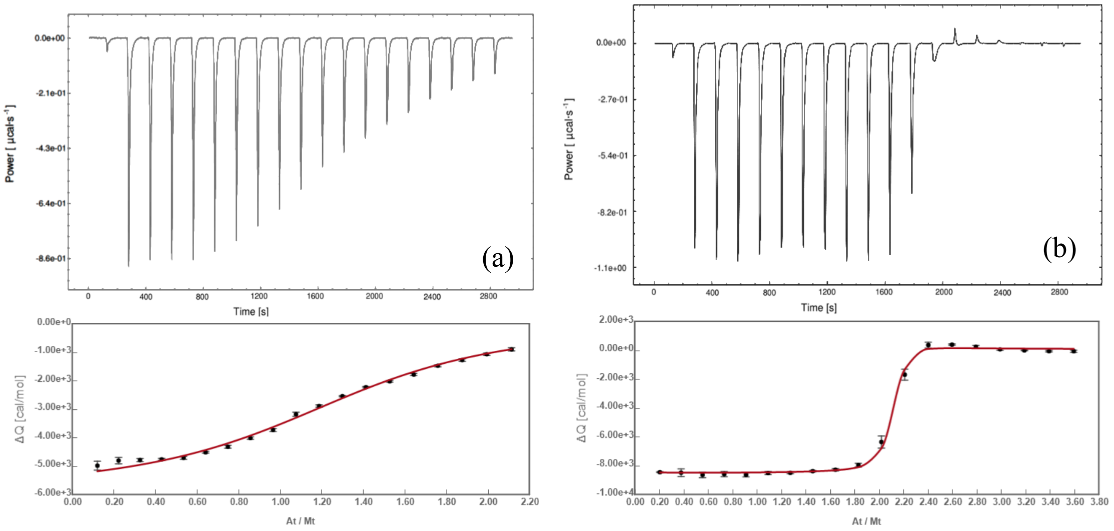

Figure S5. Representative enthalpograms for the titration of (a) CB[7] (0.10 mM) with complex **1c** (1.0 mM), and (b) CB[8] (0.050 mM) with complex **1c** (0.85 mM), in MilliQ water at 25 ºC.

8. X-ray crystallography

A specimen of $C_{92}H_{82}Cl_2N_{38}O_{16}Pt_2$, approximate dimensions 0.090 mm x 0.100 mm x 0.100 mm, was used for the X-ray crystallographic analysis. The X-ray intensity data were measured. The integration of the data using a triclinic unit cell yielded a total of 264585 reflections to a maximum θ angle of 27.00° (0.78 Å resolution), of which 47500 were independent (average redundancy 5.570, completeness = 90.6%, R_{int} = 5.81%, R_{sig} = 5.19%) and 39282 (82.70%) were greater than 2σ(F^2). The final cell constants of a = 17.9543(13) Å, b = 20.7821(16) Å, c = 33.976(3) Å, β = 82.949(4)°, volume = 12580.9(16) $Å^3$, are based upon the refinement of the XYZ-centroids of reflections above 20 σ(I). The calculated minimum and maximum transmission coefficients (based on crystal size) are 0.8000 and 0.8210. The structure was solved and refined using the Bruker SHELXTL Software Package, using the space group P -1, with Z = 4 for the formula unit, $C_{92}H_{82}Cl_2N_{38}O_{16}Pt_2$. The final anisotropic full-matrix least-squares refinement on F^2 with 2633 variables converged at R1 = 10.11%, for the observed data and wR2 = 25.54% for all data. The goodness-of-fit was 1.105. The largest peak in the final difference electron density synthesis was 3.504 $e^-/Å^3$ and the largest hole was -4.534 $e^-/Å^3$ with an RMS deviation of 0.203 $e^-/Å^3$. On the basis of the final model, the calculated density was 1.287 g/cm^3 and F(000), 4872 e^-.

Identification code	s4624
Chemical formula	$C_{92}H_{82}Cl_2N_{38}O_{16}Pt_2$
	$C_{48}H_{48}N_{32}O_{16}$, $2(C_{22}H_{17}ClN_3Pt)$ [+ solvent]
Formula weight	2437.03 g/mol
Temperature	173(2) K
Wavelength	0.71073 Å
Crystal size	0.090 x 0.100 x 0.100 mm
Crystal system	triclinic
Space group	P -1
Unit cell dimensions	a = 17.9543(13) Å α = 89°
	b = 20.7821(16) Å β = 82.949(4)°
	c = 33.976(3) Å γ = 90°
Volume	12580.9(16) $Å^3$
Z	4
Density (calculated)	1.287 g/cm^3
Abs coefficient	2.331 mm^{-1}
F(000)	4872

Theta range for data collection	1.76 to 27.00°
Index ranges	$-23 \le h \le 23$, $-24 \le k \le 25$, $-44 \le l \le 44$
Reflections collected	264585
Independent reflections	47500 [R(int) = 0.0581]
Max. and min. transmission	0.8210 and 0.8000
Structure solution technique	direct methods
Structure solution program	SHELXS-97 (Sheldrick 2008)
Refinement method	Full-matrix least-squares on F^2
Refinement program	SHELXL-2014 (Sheldrick 2014)
Function minimized	$\Sigma w(F_o^2 - F_c^2)^2$
Data / restraints / parameters	47500 / 10 / 2633
Goodness-of-fit on F2	1.105
Δ/σmax	0.002
Final R indices	39282 data; I>2σ(I) R1 = 0.1011, wR2 = 0.2486
	all data R1 = 0.1145, wR2 = 0.2554
Weighting scheme	$w=1/[\sigma^2(F_o^2)+(0.0699P)^2+328.0372P]$
	where $P=(F_o^2+2F_c^2)/3$
Largest diff. peak and hole	3.504 and -4.534 $eÅ^{-3}$
R.M.S. deviation from mean	0.203 $eÅ^{-3}$

	x/a	y/b	z/c	U(eq)
O1	0.5124(6)	0.1879(5)	0.7403(3)	0.055(3)
C1	0.5474(8)	0.2286(7)	0.7541(4)	0.041(3)
N1	0.5861(6)	0.2224(6)	0.7872(3)	0.040(3)
N2	0.5588(7)	0.2906(6)	0.7396(3)	0.046(3)
C2	0.6286(8)	0.2794(8)	0.7929(4)	0.048(4)
C3	0.6129(9)	0.3250(7)	0.7591(4)	0.048(4)
N3	0.5852(6)	0.3818(6)	0.7804(3)	0.042(3)
N4	0.6057(7)	0.3146(6)	0.8279(3)	0.040(3)
C4	0.5877(9)	0.3760(9)	0.8190(5)	0.058(5)
O2	0.5683(7)	0.4189(5)	0.8444(3)	0.059(3)
C5	0.5403(10)	0.3081(8)	0.7000(4)	0.054(4)
C6	0.5800(8)	0.4437(7)	0.7597(4)	0.046(4)
C7	0.4243(9)	0.3521(7)	0.6809(4)	0.043(4)
O3	0.3989(7)	0.3016(5)	0.6693(3)	0.061(3)
N5	0.4889(8)	0.3596(6)	0.6994(3)	0.048(3)
N6	0.3956(6)	0.4121(6)	0.6744(3)	0.040(3)
C8	0.5080(8)	0.4257(7)	0.7007(4)	0.044(3)
C9	0.4422(8)	0.4641(7)	0.6870(4)	0.042(3)
N7	0.4106(6)	0.4964(6)	0.7223(3)	0.036(3)
N8	0.5130(6)	0.4487(6)	0.7404(3)	0.042(3)
C10	0.4544(8)	0.4925(7)	0.7518(4)	0.044(3)
O4	0.4485(6)	0.5208(6)	0.7828(3)	0.059(3)
C11	0.3348(7)	0.4251(7)	0.6514(3)	0.038(3)
C12	0.3623(8)	0.5526(7)	0.7200(4)	0.044(3)
C13	0.2008(8)	0.4154(7)	0.6750(4)	0.043(3)
O5	0.1961(7)	0.3607(5)	0.6612(4)	0.070(4)
N9	0.2691(6)	0.4456(6)	0.6741(3)	0.041(3)
N10	0.1441(6)	0.4532(6)	0.6888(3)	0.044(3)
C14	0.2563(7)	0.5144(6)	0.6844(4)	0.034(3)
C15	0.1697(7)	0.5168(7)	0.6963(4)	0.040(3)
N11	0.1613(6)	0.5367(5)	0.7361(3)	0.042(3)
N12	0.2843(6)	0.5344(5)	0.7206(3)	0.037(3)
C16	0.2293(8)	0.5489(6)	0.7493(3)	0.038(3)
O6	0.2383(6)	0.5718(5)	0.7818(3)	0.050(3)
C17	0.0688(8)	0.4371(8)	0.6860(4)	0.050(4)
C18	0.0906(8)	0.5581(6)	0.7574(4)	0.042(3)
C19	0.0009(7)	0.3618(7)	0.7353(4)	0.039(3)
O7	0.0186(6)	0.3138(5)	0.7173(3)	0.049(2)
N13	0.0246(7)	0.4231(6)	0.7236(3)	0.048(3)
N14	0.9555(6)	0.3676(6)	0.7707(3)	0.044(3)
C20	0.9902(8)	0.4720(7)	0.7495(4)	0.042(3)
C21	0.9394(7)	0.4329(7)	0.7811(4)	0.045(4)
N15	0.9654(7)	0.4533(5)	0.8188(3)	0.044(3)
N16	0.0419(6)	0.5050(6)	0.7731(3)	0.046(3)
C22	0.0208(7)	0.4965(7)	0.8133(4)	0.040(3)
O8	0.0480(5)	0.5262(5)	0.8391(2)	0.046(2)
C23	0.9129(11)	0.3134(8)	0.7876(4)	0.059(5)

C24	0.9174(9)	0.4418(7)	0.8566(4)	0.053(4)
C25	0.9589(8)	0.2327(8)	0.8333(4)	0.044(4)
O9	0.9851(6)	0.1966(5)	0.8074(3)	0.054(3)
N17	0.9299(7)	0.2939(7)	0.8267(3)	0.046(3)
N18	0.9536(7)	0.2218(6)	0.8732(3)	0.047(3)
C26	0.8946(8)	0.3205(7)	0.8621(4)	0.047(4)
C27	0.9093(7)	0.2733(7)	0.8947(4)	0.043(3)
N19	0.9506(7)	0.3068(6)	0.9208(3)	0.047(3)
N20	0.9294(7)	0.3797(6)	0.8738(4)	0.046(3)
C28	0.9626(8)	0.3744(7)	0.9111(4)	0.044(4)
O10	0.9788(5)	0.4149(4)	0.9326(2)	0.035(2)
C29	0.9610(7)	0.1577(7)	0.8904(4)	0.044(4)
C30	0.9540(8)	0.2854(8)	0.9612(4)	0.044(4)
C31	0.0750(6)	0.1121(7)	0.9090(3)	0.035(3)
O11	0.0942(5)	0.0836(5)	0.8776(3)	0.047(2)
N21	0.0205(6)	0.1554(5)	0.9156(3)	0.039(3)
N22	0.1099(6)	0.1040(6)	0.9420(3)	0.039(3)
C32	0.0059(7)	0.1735(7)	0.9557(4)	0.043(4)
C33	0.0741(7)	0.1426(7)	0.9754(4)	0.041(3)
N23	0.1168(5)	0.1981(6)	0.9848(3)	0.036(3)
N24	0.0135(6)	0.2405(6)	0.9642(3)	0.043(3)
C34	0.0774(8)	0.2536(7)	0.9815(4)	0.043(4)
O12	0.0970(5)	0.3072(5)	0.9911(3)	0.049(3)
C35	0.1641(9)	0.0561(8)	0.9477(5)	0.053(4)
C36	0.1726(5)	0.1936(8)	0.0124(4)	0.043(2)
C37	0.2965(7)	0.0582(6)	0.9204(4)	0.034(3)
O13	0.2903(5)	0.0279(5)	0.8909(3)	0.043(2)
N25	0.2408(6)	0.0795(6)	0.9477(3)	0.046(3)
N26	0.3636(6)	0.0803(6)	0.9316(3)	0.038(3)
C38	0.2646(6)	0.1055(8)	0.9818(5)	0.043(2)
C39	0.3541(8)	0.1100(8)	0.9706(4)	0.044(4)
N27	0.3678(5)	0.1773(6)	0.9705(4)	0.047(3)
N28	0.2397(5)	0.1751(6)	0.9903(4)	0.043(2)
C40	0.3075(7)	0.2137(8)	0.9842(4)	0.041(3)
O14	0.3081(5)	0.2693(5)	0.9921(3)	0.053(3)
C41	0.4359(8)	0.0564(7)	0.9133(4)	0.045(3)
C42	0.4446(8)	0.2003(8)	0.9704(4)	0.054(4)
C43	0.4992(7)	0.1098(7)	0.8524(3)	0.054(4)
O15	0.4726(6)	0.0760(5)	0.8288(3)	0.048(2)
N29	0.4806(6)	0.1063(6)	0.8936(3)	0.039(3)
N30	0.5530(6)	0.1555(6)	0.8439(3)	0.037(3)
C44	0.5272(7)	0.1475(7)	0.9138(4)	0.041(3)
C45	0.5805(6)	0.1784(6)	0.8819(3)	0.029(3)
N31	0.5727(7)	0.2467(6)	0.8870(3)	0.043(3)
N32	0.4887(6)	0.2044(6)	0.9325(3)	0.039(3)
C46	0.5184(7)	0.2608(7)	0.9165(4)	0.037(3)
O16	0.5009(5)	0.3156(5)	0.9290(3)	0.048(2)
C47	0.5959(8)	0.1622(8)	0.8044(4)	0.045(4)
C48	0.6227(8)	0.2960(7)	0.8678(4)	0.040(3)
C49	0.1838(9)	0.5563(7)	0.5766(4)	0.047(4)
O17	0.1890(6)	0.5249(5)	0.6074(3)	0.054(3)
N33	0.1186(5)	0.5806(5)	0.5663(3)	0.036(2)
N34	0.2406(6)	0.5717(6)	0.5475(3)	0.0418(12)
C50	0.1297(8)	0.6098(7)	0.5257(4)	0.042(3)
C51	0.2156(7)	0.5999(7)	0.5127(4)	0.044(4)
N35	0.2401(6)	0.6661(6)	0.5049(3)	0.0418(12)
N36	0.1207(6)	0.6806(5)	0.5250(3)	0.037(3)
C52	0.1826(8)	0.7094(8)	0.5090(4)	0.043(3)
O18	0.1897(5)	0.7684(5)	0.5007(3)	0.050(3)
C53	0.3146(8)	0.5464(7)	0.5480(4)	0.0418(12)
C54	0.3050(7)	0.6869(8)	0.4820(4)	0.0418(12)
C55	0.4035(7)	0.5993(6)	0.5868(3)	0.034(3)
O19	0.3856(6)	0.5695(5)	0.6180(3)	0.048(2)
N37	0.3694(6)	0.5953(6)	0.5529(3)	0.0418(12)
N38	0.4634(6)	0.6410(6)	0.5794(3)	0.039(3)
C56	0.4118(7)	0.6288(8)	0.5201(4)	0.043(4)
C57	0.4795(8)	0.6591(7)	0.5382(4)	0.043(4)
N39	0.4717(7)	0.7273(6)	0.5304(3)	0.048(3)
N40	0.3719(6)	0.6860(6)	0.5054(3)	0.0418(12)
C58	0.4117(8)	0.7410(9)	0.5102(4)	0.044(4)
O20	0.3966(6)	0.7950(6)	0.4970(4)	0.062(3)
C59	0.5202(9)	0.6429(8)	0.6081(4)	0.052(4)
C60	0.5305(8)	0.7744(8)	0.5349(4)	0.047(4)
C61	0.5122(8)	0.7154(8)	0.6647(4)	0.046(4)
O21	0.4824(6)	0.6775(5)	0.6894(3)	0.057(3)
N41	0.5260(6)	0.7051(6)	0.6237(3)	0.042(3)
N42	0.5349(8)	0.7750(6)	0.6718(3)	0.056(4)
C62	0.5676(9)	0.7584(7)	0.6037(4)	0.0503(14)
C63	0.5760(9)	0.8057(7)	0.6371(4)	0.0503(14)
N43	0.5416(7)	0.8663(6)	0.6221(3)	0.0503(14)
N44	0.5308(7)	0.7962(6)	0.5756(3)	0.0503(14)
C64	0.5230(9)	0.8556(8)	0.5872(4)	0.0503(14)
O22	0.4910(6)	0.9008(6)	0.5678(3)	0.0503(14)
C65	0.5478(12)	0.7945(8)	0.7118(5)	0.067(5)
C66	0.5473(10)	0.9273(7)	0.6394(5)	0.067(5)
C67	0.4572(9)	0.8452(7)	0.7607(4)	0.051(4)
O23	0.4394(7)	0.7960(5)	0.7806(3)	0.061(3)
N45	0.5021(8)	0.8476(6)	0.7264(4)	0.052(3)
N46	0.4378(7)	0.9078(6)	0.7722(3)	0.047(3)
C68	0.5216(8)	0.9131(8)	0.7142(4)	0.048(4)
C69	0.4730(8)	0.9544(8)	0.7452(4)	0.049(4)
N47	0.4207(6)	0.9874(6)	0.7208(3)	0.041(3)
N48	0.4993(7)	0.9353(6)	0.6771(3)	0.044(3)
C70	0.4422(9)	0.9809(7)	0.6817(4)	0.041(3)
O24	0.4174(6)	0.0073(5)	0.6542(3)	0.050(3)
C71	0.3968(9)	0.9231(8)	0.8116(4)	0.049(4)
C72	0.3794(7)	0.0443(6)	0.7364(4)	0.038(3)
C73	0.2621(8)	0.9115(8)	0.8284(4)	0.039(3)
O25	0.2644(7)	0.8552(6)	0.8424(3)	0.065(3)
N49	0.3234(7)	0.9447(6)	0.8094(3)	0.043(3)
N50	0.2006(6)	0.9496(5)	0.8291(3)	0.037(3)
C74	0.3066(8)	0.0104(6)	0.8025(3)	0.035(3)
C75	0.2213(8)	0.0136(7)	0.8162(3)	0.038(3)
N51	0.1891(6)	0.0360(5)	0.7810(3)	0.037(3)
N52	0.3103(6)	0.0279(5)	0.7597(3)	0.038(3)
C76	0.2412(8)	0.0444(7)	0.7488(4)	0.039(3)
O26	0.2311(6)	0.0658(5)	0.7162(3)	0.053(3)
C77	0.1308(8)	0.9314(7)	0.8510(4)	0.042(3)
C78	0.1145(6)	0.0609(6)	0.7820(3)	0.032(3)
C79	0.0411(8)	0.8658(7)	0.8201(3)	0.053(3)
O27	0.0665(6)	0.8136(6)	0.8311(3)	0.053(3)
N53	0.0698(7)	0.9235(6)	0.8263(3)	0.044(3)
N54	0.9778(7)	0.8706(6)	0.8018(3)	0.048(3)
C80	0.9591(7)	0.9393(7)	0.7988(4)	0.038(3)
C81	0.0227(8)	0.9762(6)	0.8156(4)	0.036(3)
N55	0.9603(7)	0.9639(6)	0.7596(3)	0.045(3)
N56	0.0567(7)	0.0116(6)	0.7806(3)	0.046(3)
C82	0.0126(8)	0.0081(6)	0.7508(4)	0.039(3)
O28	0.0252(5)	0.0411(5)	0.7193(3)	0.047(2)
C83	0.9284(9)	0.8180(7)	0.7990(4)	0.051(4)
C84	0.9005(9)	0.9541(7)	0.7369(4)	0.049(4)
C85	0.9338(7)	0.7393(8)	0.7455(4)	0.037(3)
O29	0.9658(6)	0.6970(5)	0.7609(3)	0.048(3)
N57	0.9207(7)	0.8024(5)	0.7579(3)	0.040(3)
N58	0.9031(6)	0.7346(6)	0.7092(3)	0.042(3)
C86	0.8619(7)	0.7905(7)	0.7000(4)	0.036(3)
C87	0.8726(8)	0.8369(7)	0.7357(4)	0.039(3)
N59	0.8953(6)	0.8274(6)	0.6663(3)	0.041(3)
N60	0.9014(6)	0.8945(5)	0.7153(3)	0.035(2)
C88	0.9084(6)	0.8916(7)	0.6747(3)	0.034(3)
O30	0.9268(6)	0.9329(5)	0.6525(3)	0.049(3)
C89	0.8930(8)	0.6735(7)	0.6921(4)	0.043(3)
C90	0.8798(7)	0.8108(8)	0.6269(4)	0.045(4)
C91	0.9872(7)	0.6193(7)	0.6453(4)	0.038(3)
O31	0.0146(5)	0.5865(5)	0.6689(3)	0.043(2)
N61	0.9359(5)	0.6687(6)	0.6529(3)	0.039(3)
N62	0.0033(6)	0.6136(5)	0.6039(3)	0.040(3)
C92	0.9113(5)	0.6945(5)	0.6170(3)	0.025(3)
C93	0.9608(7)	0.6607(7)	0.5833(3)	0.040(3)
N63	0.0055(5)	0.7129(5)	0.5647(3)	0.034(2)
N64	0.9226(6)	0.7608(5)	0.6091(3)	0.043(3)
C94	0.9828(7)	0.7708(7)	0.5809(4)	0.037(3)
O32	0.0100(5)	0.8228(5)	0.5710(3)	0.046(2)
C95	0.0459(7)	0.5617(6)	0.5851(4)	0.044(3)
C96	0.0465(7)	0.7092(8)	0.5252(3)	0.047(4)
Pt1	0.21218(3)	0.59810(3)	0.99778(2)	0.03852(14)
Cl1	0.2401(2)	0.66857(19)	0.04564(11)	0.0534(10)
N65	0.2141(6)	0.5154(6)	0.0291(3)	0.044(3)
C97	0.2250(7)	0.5102(9)	0.0688(4)	0.051(4)
C98	0.2260(9)	0.4500(10)	0.0856(5)	0.062(5)
C99	0.2177(12)	0.3977(10)	0.0655(6)	0.076(6)
C100	0.2073(8)	0.4005(8)	0.0250(4)	0.047(4)
C101	0.2037(7)	0.4618(8)	0.0085(4)	0.045(4)
C102	0.1907(7)	0.4742(7)	0.9669(4)	0.038(3)
C103	0.1801(7)	0.4300(8)	0.9375(4)	0.043(3)
C104	0.1726(8)	0.4489(9)	0.8989(5)	0.061(5)
C105	0.1716(8)	0.5170(7)	0.8924(4)	0.043(3)
C106	0.1816(7)	0.5610(7)	0.9214(4)	0.037(3)
N66	0.1906(6)	0.5354(7)	0.9585(4)	0.047(3)
C107	0.1852(8)	0.6299(8)	0.9186(5)	0.049(4)
C108	0.1724(9)	0.6675(9)	0.8861(5)	0.055(4)
C109	0.1793(10)	0.7320(11)	0.8873(7)	0.077(6)
C110	0.1936(10)	0.7656(11)	0.9188(6)	0.073(5)
C111	0.2053(8)	0.7233(8)	0.9538(5)	0.055(4)
N67	0.2016(6)	0.6593(6)	0.9526(3)	0.045(3)
C112	0.1653(8)	0.4021(10)	0.8677(4)	0.067(6)
C113	0.1871(8)	0.4199(10)	0.8271(5)	0.061(5)
C114	0.1849(9)	0.3778(10)	0.7987(5)	0.069(5)
C115	0.1611(7)	0.3155(10)	0.8071(4)	0.055(4)
C116	0.1366(8)	0.2992(9)	0.8459(5)	0.058(4)
C117	0.1396(8)	0.3410(8)	0.8752(5)	0.057(4)
C118	0.1586(11)	0.2659(12)	0.7728(7)	0.100(8)
Pt2	0.39593(2)	0.60704(3)	0.95551(2)	0.03215(13)
Cl2	0.4255(2)	0.7026(2)	0.98302(12)	0.0589(11)
N68	0.4067(5)	0.5472(5)	0.0015(3)	0.032(2)
C119	0.4215(6)	0.5631(8)	0.0378(4)	0.044(4)
C120	0.4249(7)	0.5163(9)	0.0664(4)	0.052(4)
C121	0.4161(8)	0.4530(9)	0.0574(4)	0.048(4)
C122	0.4027(7)	0.4333(8)	0.0206(4)	0.052(4)
C123	0.3993(7)	0.4832(7)	0.9918(4)	0.035(3)
C124	0.3838(7)	0.4718(7)	0.9502(4)	0.036(3)
C125	0.3784(7)	0.4120(6)	0.9319(4)	0.037(3)
C126	0.3700(6)	0.4138(7)	0.8911(4)	0.040(4)
C127	0.3639(7)	0.4721(7)	0.8720(4)	0.038(3)
C128	0.3707(6)	0.5283(6)	0.8905(3)	0.029(3)
N69	0.3811(5)	0.5264(5)	0.9300(3)	0.031(2)
C129	0.3663(8)	0.5944(7)	0.8760(4)	0.041(3)
C130	0.3494(9)	0.6093(7)	0.8380(4)	0.043(3)
C131	0.3497(11)	0.6703(9)	0.8266(5)	0.067(5)
C132	0.3601(13)	0.7230(9)	0.8519(6)	0.083(6)
C133	0.3754(9)	0.7067(7)	0.8899(4)	0.046(4)
N70	0.3784(6)	0.6440(5)	0.9018(3)	0.036(3)
C134	0.3657(7)	0.3528(7)	0.8700(4)	0.039(3)
C135	0.3879(9)	0.3468(8)	0.8316(5)	0.057(4)
C136	0.3791(10)	0.2934(9)	0.8081(5)	0.062(5)
C137	0.3436(10)	0.2371(9)	0.8304(7)	0.072(6)
C138	0.3230(11)	0.2429(9)	0.8691(7)	0.073(5)
C139	0.3334(10)	0.2994(8)	0.8896(5)	0.058(4)
C140	0.3313(16)	0.1748(11)	0.8082(8)	0.128(11)
Pt3	0.10417(2)	0.10290(2)	0.54602(2)	0.02874(12)
Cl3	0.0830(2)	0.1853(2)	0.50367(12)	0.0569(10)
N71	0.0987(5)	0.0262(6)	0.5094(3)	0.037(3)
C141	0.0876(7)	0.0292(9)	0.4712(3)	0.048(4)
C142	0.0852(7)	0.9711(8)	0.4494(4)	0.049(4)
C143	0.0929(7)	0.9153(9)	0.4688(4)	0.053(4)
C144	0.1050(6)	0.9087(7)	0.5095(4)	0.044(4)
C145	0.1075(6)	0.9688(7)	0.5281(4)	0.033(3)
C146	0.1165(7)	0.9711(7)	0.5718(4)	0.037(3)
C147	0.1220(7)	0.9222(7)	0.5992(4)	0.039(3)
C148	0.1325(8)	0.9383(7)	0.6374(4)	0.045(4)
C149	0.1357(8)	0.0006(7)	0.6481(4)	0.043(3)
C150	0.1283(7)	0.0500(6)	0.6202(3)	0.034(3)
N72	0.1206(5)	0.0322(5)	0.5824(3)	0.031(2)
C151	0.1240(7)	0.1160(7)	0.6276(3)	0.040(3)
C152	0.1286(11)	0.1478(9)	0.6625(4)	0.067(5)
C153	0.1250(10)	0.2139(9)	0.6640(5)	0.063(5)
C154	0.1110(9)	0.2472(8)	0.6334(5)	0.059(5)
C155	0.1061(8)	0.2174(7)	0.5982(5)	0.050(4)
N73	0.1115(5)	0.1555(5)	0.5940(3)	0.031(2)
C156	0.1348(7)	0.8862(7)	0.6681(3)	0.041(3)
C157	0.1752(10)	0.8967(8)	0.7002(4)	0.058(4)
C158	0.1791(13)	0.8478(10)	0.7294(5)	0.083(6)
C159	0.1451(10)	0.7856(8)	0.7262(5)	0.064(5)
C160	0.1093(9)	0.7787(8)	0.6925(5)	0.050(4)
C161	0.1054(9)	0.8270(8)	0.6649(4)	0.052(4)
C162	0.1519(11)	0.7330(9)	0.7546(5)	0.066(5)
Pt4	0.28950(3)	0.09659(3)	0.48962(2)	0.04422(16)
Cl4	0.2747(2)	0.1833(2)	0.44858(13)	0.0651(11)

N74	0.2816(5)	0.0261(6)	0.4476(3)	0.042(3)
C163	0.2678(9)	0.0390(10)	0.4106(4)	0.062(5)
C164	0.2657(10)	0.9864(12)	0.3843(5)	0.085(7)
C165	0.2732(9)	0.9233(10)	0.3984(4)	0.060(5)
C166	0.2891(8)	0.9136(8)	0.4379(4)	0.048(4)
C167	0.2875(8)	0.9678(9)	0.4611(4)	0.054(4)
C168	0.3001(7)	0.9643(7)	0.5044(4)	0.040(3)
C169	0.3046(6)	0.9098(7)	0.5269(4)	0.035(3)
C170	0.3114(7)	0.9156(7)	0.5660(4)	0.037(3)
C171	0.3169(7)	0.9725(7)	0.5847(5)	0.043(3)
C172	0.3103(7)	0.0297(7)	0.5581(5)	0.049(4)
N75	0.3020(6)	0.0228(6)	0.5211(3)	0.045(3)
C173	0.3108(7)	0.0974(9)	0.5703(5)	0.057(4)
C174	0.3169(10)	0.1186(9)	0.6090(5)	0.066(5)
C175	0.3110(11)	0.1846(11)	0.6160(6)	0.078(6)
C176	0.2992(12)	0.2285(13)	0.5856(7)	0.097(8)
C177	0.2962(9)	0.2031(9)	0.5489(6)	0.069(5)
N76	0.3005(6)	0.1419(7)	0.5405(4)	0.050(3)
C178	0.3093(9)	0.8551(7)	0.5941(4)	0.045(4)
C179	0.3413(8)	0.8548(8)	0.6289(4)	0.053(4)
C180	0.3359(10)	0.8028(8)	0.6535(4)	0.059(4)
C181	0.3014(9)	0.7451(9)	0.6446(4)	0.057(4)
C182	0.2682(12)	0.7438(9)	0.6084(6)	0.080(6)
C183	0.2746(10)	0.7991(9)	0.5823(5)	0.069(5)
C184	0.2936(14)	0.6875(10)	0.6720(6)	0.089(7)

9. Computational details

Complexes and assemblies **1b**, CB[7]·**1b**, **1b**$_2$, CB[8]·**1b**$_2$ and their hydrides were optimized with the semiempirical tight-binding method GFN2-xTB[7,8] in conjunction with the GBSA solvation model.[9,10] Enthalpic and entropic contributions at 25 ºC ($\Delta G_{T,\mathrm{xTB}}$) and free energies of solvation ($\Delta G_{\mathrm{solv,xTB}}$) were also calculated with this method. The complexes and assemblies were then reoptimized by density functional theory with the TURBOMOLE[11–14] suite of programs (version 7.2.1) at the B97-3c/def2-mTZVP level with COSMO[15,16] solvation parameters to extract the electronic contribution at 0 K ($\Delta E_{\mathrm{B97\text{-}3c}}$). The m4 grid size was used and convergence criteria were 10^{-6} hartree. The relative stability (ΔG) of the four assemblies was calculated using equation (3).

$$\Delta G = \Delta E_{\mathrm{B97-3c}} + \Delta G_{T,\mathrm{xTB}} + \Delta G_{\mathrm{solv,xTB}} \qquad (3)$$

Table S3. Electronic, vibrational and solvation terms [kcal/mol] calculated for PCET processes.

PCET process	$\Delta E_{\mathrm{B97\text{-}3c}}$	$\Delta G_{T,\mathrm{xTB}}$	$\Delta G_{\mathrm{solv,xTB}}$	ΔG
$\mathbf{1b}^{+} + \mathrm{H}\cdot \rightarrow [\mathbf{1b}\text{–H}]^{+}$	–21.8	8.3	0.2	–13.2
$\mathrm{CB[7]}\cdot\mathbf{1b}^{+} + \mathrm{H}\cdot \rightarrow [\mathrm{CB[7]}\cdot\mathbf{1b}\text{–H}]^{+}$	–20.7	11.0	–3.9	–13.6
$\mathbf{1b}_2^{2+} + \mathrm{H}\cdot \rightarrow [\mathbf{1b_2}\text{–H}]^{2+}$	–26.8	10.1	–1.3	–18.0
$\mathrm{CB[8]}\cdot\mathbf{1b}_2^{2+} + \mathrm{H}\cdot \rightarrow [\mathrm{CB[8]}\cdot\mathbf{1b_2}\text{–H}]^{2+}$	–28.7	9.6	–1.1	–20.2

10. Coordinates of optimized structures

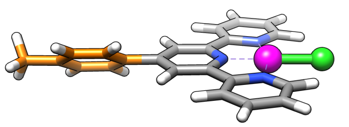

1b

Cl	11.28162	15.09803	33.02178
N	11.70180	11.85901	33.50855
C	12.22586	12.26030	34.67188
H	12.23569	13.32703	34.83444
C	12.71873	11.35514	35.59380
H	13.13374	11.71864	36.52153
C	12.66830	10.00185	35.30584
H	13.04713	9.27471	36.00909
C	12.12489	9.58532	34.10087
H	12.07736	8.53428	33.85988
C	11.64182	10.52141	33.20383
C	11.04468	10.20380	31.90477
C	10.86100	8.96295	31.32656
H	11.19544	8.07233	31.83493
C	10.27822	8.85405	30.05474
C	9.89439	10.03389	29.39921
H	9.41388	9.97283	28.43541
C	10.09427	11.26133	29.99998
N	10.65864	11.30517	31.22578
C	9.75664	12.59049	29.48574
C	9.15548	12.83507	28.26384
H	8.90002	12.01107	27.61517
C	8.88265	14.13808	27.87849
H	8.41246	14.33380	26.92592
C	9.21848	15.17926	28.72706
H	9.02250	16.20720	28.46255
C	9.81796	14.89478	29.94031
H	10.10389	15.65888	30.64659
N	10.08081	13.63740	30.31312
C	10.07865	7.54906	29.42942
C	10.21936	7.38507	28.04806
H	10.52068	8.22083	27.43088
C	10.04400	6.14659	27.46222
H	10.18236	6.04152	26.39434
C	9.70281	5.02774	28.22202
C	9.55779	5.19577	29.59945
H	9.28655	4.34642	30.21240
C	9.74808	6.42676	30.19555
H	9.59398	6.52437	31.26185
C	9.47435	3.69853	27.58014
H	9.74719	2.88029	28.24243
H	10.03914	3.59536	26.65701
H	8.41988	3.57092	27.32959
Pt	10.93941	13.03826	32.04788

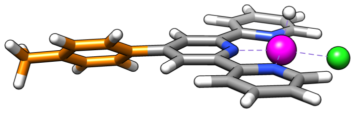

1b-H

Cl	10.93882	15.08091	33.17740	C	9.99847	10.03688	29.35714	C	10.25007	7.36714	28.02627
N	11.65380	11.85005	33.53987	H	9.52155	9.97173	28.39178	H	10.56487	8.18507	27.39203
C	12.14137	12.25020	34.72630	C	10.16976	11.26667	29.95851	C	10.00727	6.13138	27.45709
H	12.08488	13.30983	34.92036	N	10.70819	11.30468	31.20672	H	10.10894	6.01243	26.38659
C	12.66937	11.35168	35.62523	C	9.80714	12.57880	29.45608	C	9.64142	5.03434	28.23573
H	13.03979	11.71003	36.57346	C	9.26620	12.83422	28.20349	C	9.54651	5.21903	29.61585
C	12.71748	10.00141	35.29156	H	9.09716	12.01623	27.51973	H	9.25925	4.38651	30.24472
H	13.13740	9.28103	35.97785	C	8.94476	14.12659	27.83793	C	9.79996	6.44656	30.19372
C	12.22419	9.58908	34.06946	H	8.52046	14.32536	26.86495	H	9.67566	6.55961	31.26232
H	12.25747	8.54639	33.79198	C	9.17542	15.16561	28.73459	C	9.33531	3.70932	27.61752
C	11.68353	10.51532	33.18812	H	8.93236	16.18732	28.48601	H	9.74343	2.88973	28.20574
C	11.12287	10.20263	31.88694	C	9.72827	14.88575	29.96353	H	9.72958	3.63407	26.60769
C	10.96008	8.96786	31.29330	H	9.94016	15.64633	30.69857	H	8.25698	3.55313	27.55998
H	11.29239	8.07724	31.80320	N	10.03311	13.62797	30.32455	Pt	10.96077	13.04699	32.04322
C	10.38044	8.85781	30.01840	C	10.14999	7.55064	29.40835	H	12.41259	13.42597	31.52573

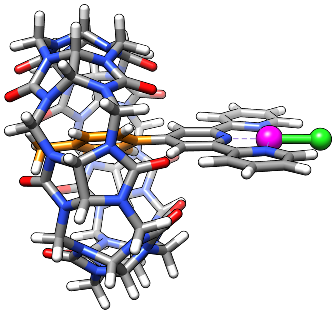

CB[7]·1b

Cl	10.34490	14.38939	33.34831	C	12.01496	7.32888	23.56762	N	4.59760	4.77991	30.35276
N	11.22443	11.21225	33.72582	N	11.20783	6.13011	23.61034	C	4.76027	5.79894	31.34354
C	11.64883	11.64132	34.91918	C	11.94649	5.02366	24.01329	C	5.48678	5.06519	32.50154
H	11.49424	12.69094	35.11643	H	14.20692	7.22996	23.93182	N	5.82446	3.78919	31.92552
C	12.24511	10.78392	35.82402	H	12.24801	7.60100	22.53348	C	5.19431	3.57961	30.70411
H	12.57565	11.16813	36.77745	N	13.05946	7.63603	25.64227	H	3.78570	6.20550	31.63554
C	12.40538	9.45150	35.48183	N	11.50685	8.46506	24.27688	H	4.85771	4.92891	33.38807
H	12.87043	8.75925	36.16918	C	12.08640	8.61382	25.52311	N	5.66372	6.88145	31.01429
C	11.96697	9.00282	34.24715	O	11.60044	3.86696	23.95736	N	6.58470	5.93502	32.81008
H	12.07614	7.96955	33.96630	O	11.81075	9.46645	26.34248	C	6.68931	7.00495	31.94068
C	11.37204	9.89303	33.37169	C	10.00829	5.95651	22.83333	O	5.13632	2.53966	30.09286
C	10.87289	9.54432	32.04056	H	10.14227	6.39976	21.84163	O	7.49866	7.90621	32.00641
C	10.87567	8.31019	31.42114	H	9.85671	4.88490	22.73132	C	6.30094	2.67790	32.70345
H	11.30919	7.46438	31.92720	C	10.44340	9.31219	23.81445	H	5.74912	2.62014	33.64629
C	10.34139	8.17190	30.13187	H	10.56292	9.45040	22.73757	H	6.10609	1.78403	32.11690
C	9.83884	9.31411	29.49412	H	10.54441	10.27137	24.31408	C	7.39485	5.80937	33.98432
H	9.39159	9.24077	28.51757	N	14.41721	3.51297	28.31444	H	6.74724	5.54110	34.82489
C	9.86611	10.53510	30.13553	C	15.19507	4.71342	28.49162	H	7.85191	6.77595	34.17732
N	10.37014	10.60904	31.38295	C	15.00709	5.46063	27.14635	C	11.95675	4.15219	34.47237
C	9.39859	11.82725	29.63590	N	14.36207	4.49241	26.31483	H	12.18600	5.11421	34.92050
C	8.88044	12.03628	28.37197	C	14.02569	3.33221	26.99386	H	12.23165	3.35679	35.16620
H	8.78194	11.21681	27.68121	H	16.24019	4.46252	28.70428	C	3.76158	4.90470	29.19163
C	8.48733	13.31028	27.99859	H	15.95250	5.78026	26.69442	H	2.79428	5.31842	29.48841
H	8.08795	13.47405	27.00807	N	14.70797	5.64802	29.46242	H	3.62102	3.90085	28.80012
C	8.61554	14.35643	28.89737	N	14.22156	6.61850	27.51601	C	5.23967	8.01942	30.24469
H	8.32038	15.36304	28.64121	C	14.13356	6.77045	28.89227	H	5.90575	8.84355	30.48481
C	9.14226	14.10509	30.15053	O	13.53243	2.33690	26.51825	H	4.21714	8.28699	30.54016
H	9.27677	14.87232	30.89730	O	13.68532	7.72654	29.48778	C	14.49903	2.39170	29.21159
N	9.52695	12.87654	30.51249	N	12.10363	2.14035	31.89040	H	15.54397	2.21392	29.48588
C	10.28513	6.87111	29.46575	C	13.12830	2.78785	32.66725	H	14.11845	1.53058	28.66871
C	10.32591	6.78142	28.07038	C	14.16407	3.21270	31.59087	C	14.94243	5.52835	30.86922
H	10.46851	7.67688	27.48464	N	13.73190	2.51773	30.41911	H	14.83109	6.51498	31.30981
C	10.23902	5.56023	27.43235	C	12.50862	1.89298	30.58566	H	15.96841	5.17796	31.02118
H	10.27613	5.52007	26.35178	H	13.53714	2.10067	33.41489	C	11.00580	1.42014	32.47347
C	10.08413	4.37309	28.15048	H	15.19178	2.93319	31.84360	H	11.35630	0.87797	33.35879
C	10.05852	4.46365	29.54374	N	12.76846	4.03173	33.29139	H	10.65969	0.70681	31.72926
H	9.93328	3.55883	30.12189	N	14.03430	4.65693	31.57353	N	5.28621	7.84091	28.81688
C	10.15878	5.68230	30.19093	C	13.30028	5.12451	32.64022	C	4.24699	7.13695	28.08297
H	10.09219	5.71616	31.26962	O	11.92607	1.21982	29.76781	C	4.60127	7.46120	26.61167
C	9.91068	3.06673	27.44986	O	13.18298	6.28826	32.97664	N	5.49491	8.57634	26.72528
H	10.18938	2.23110	28.08385	C	14.21747	4.61861	24.89259	C	5.93847	8.76518	28.01989
H	10.49588	3.02046	26.53499	H	15.16292	4.96357	24.46681	H	3.25958	7.50104	28.38413
H	8.86543	2.93437	27.16452	H	13.98781	3.62783	24.51014	H	3.73246	7.73555	26.00453
Pt	10.36268	12.33098	32.27523	C	14.08425	7.75531	26.64747	N	4.29069	5.71080	28.12718
N	8.82269	6.50859	23.42400	H	13.82355	8.60859	27.26796	N	5.17986	6.22169	26.15133
C	8.40844	7.87088	23.28607	H	15.04647	7.94863	26.15656	C	4.88304	5.16159	26.99967
C	6.97426	7.87617	23.88998	N	7.70509	2.70042	33.00887	O	6.72256	9.61648	28.39073
N	6.81920	6.54122	24.39983	C	8.29881	3.42702	34.08575	O	5.06142	3.98753	26.77920
C	7.88838	5.71804	24.07365	C	9.76137	2.90891	34.10509	C	5.54903	5.98482	24.78367
H	8.43552	8.17374	22.23451	N	9.87858	2.23987	32.83230	H	5.61031	4.90641	24.65935
H	6.19640	8.09448	23.15120	C	8.63910	2.03130	32.23661	H	4.77507	6.38217	24.11706
N	9.11136	8.84259	24.09395	H	7.75946	3.23954	35.01912	C	5.90727	9.40703	25.62756
N	7.03126	8.93340	24.86737	H	9.96436	2.21557	34.92707	H	6.19401	10.37298	26.03262
C	8.27408	9.52115	24.95295	N	8.47280	4.85545	33.88816	H	5.05170	9.53477	24.96152
O	7.97063	4.52971	24.27584	N	10.53360	4.10899	34.27079				
O	8.56705	10.49195	25.62445	C	9.77541	5.25169	34.11288				
N	13.17153	5.49812	24.44797	O	8.41735	1.36107	31.25610				
C	13.26259	6.92382	24.39292	O	10.17656	6.39588	34.19595				

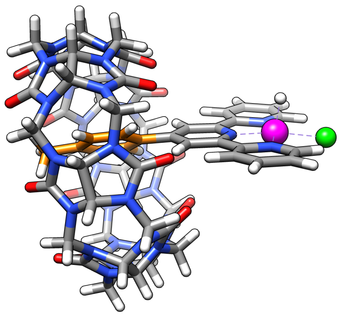

CB[7]·1b-H

Cl	9.99357	14.32182	33.51729	N	13.19741	5.43473	24.42185	C	9.78862	5.29315	34.08985
N	11.13462	11.19232	33.77603	C	13.29731	6.85935	24.35059	O	8.38860	1.40248	31.25219
C	11.54344	11.61305	34.98422	C	12.03771	7.26532	23.54362	O	10.19516	6.43556	34.16809
H	11.34054	12.64840	35.20892	N	11.22413	6.07182	23.61022	N	4.57899	4.84101	30.37831
C	12.17511	10.76317	35.86243	C	11.96302	4.96434	24.00989	C	4.74908	5.85833	31.36987
H	12.48070	11.13545	36.82876	H	14.23524	7.15360	23.86905	C	5.48397	5.12263	32.52152
C	12.40880	9.44500	35.48157	H	12.25447	7.52693	22.50315	N	5.81286	3.84562	31.94290
H	12.91183	8.76168	36.15050	N	13.12211	7.58610	25.59522	C	5.17259	3.63806	30.72643
C	11.99738	9.00965	34.23749	N	11.54913	8.41083	24.25158	H	3.77653	6.26429	31.66945
H	12.16361	7.99263	33.92629	C	12.15576	8.57094	25.48373	H	4.86223	4.98800	33.41351
C	11.34854	9.88679	33.38126	O	11.60932	3.80925	23.96836	N	5.64986	6.94150	31.03716
C	10.86675	9.55151	32.05412	O	11.90503	9.43714	26.29647	N	6.58672	5.99025	32.82119
C	10.89526	8.33064	31.41110	C	10.01097	5.89858	22.85455	C	6.68227	7.06387	31.95539
H	11.34294	7.48545	31.90600	H	10.13381	6.32669	21.85451	O	5.10476	2.59787	30.11673
C	10.36401	8.19906	30.11889	H	9.84798	4.82707	22.76994	O	7.48997	7.96721	32.01817
C	9.84477	9.34017	29.48959	C	10.48768	9.26517	23.79870	C	6.29139	2.73213	32.71661
H	9.40294	9.26881	28.51015	H	10.59458	9.39864	22.71982	H	5.74803	2.67722	33.66455
C	9.83542	10.55452	30.14256	H	10.60329	10.22501	24.29408	H	6.08694	1.83908	32.13201
N	10.32418	10.61333	31.40604	N	14.44070	3.46564	28.29117	C	7.40886	5.85954	33.98669
C	9.33828	11.83000	29.66373	C	15.23048	4.65942	28.46108	H	6.76934	5.59233	34.83388
C	8.84558	12.05959	28.38892	C	15.05475	5.39774	27.10884	H	7.87155	6.82420	34.17607
H	8.80302	11.25543	27.67440	N	14.39767	4.43094	26.28441	C	11.96726	4.18429	34.43874
C	8.40718	13.32157	28.04064	C	14.04949	3.27903	26.97147	H	12.20340	5.14987	34.87565
H	8.02656	13.49779	27.04509	H	16.27229	4.39910	28.67881	H	12.23986	3.39465	35.14029
C	8.46729	14.35192	28.97428	H	16.00530	5.69988	26.65559	C	3.73994	4.96924	29.21954
H	8.13056	15.35011	28.73745	N	14.75045	5.60711	29.42277	H	2.77919	5.39695	29.51741
C	8.97754	14.09535	30.22651	N	14.28580	6.56954	27.46739	H	3.58543	3.96480	28.83474
H	9.06643	14.84958	30.99253	C	14.19382	6.73322	28.84192	C	5.22666	8.07407	30.25987
N	9.40130	12.86819	30.57041	O	13.54764	2.28482	26.50232	H	5.88934	8.90118	30.49944
C	10.31884	6.90167	29.44562	O	13.75689	7.69994	29.42830	H	4.20169	8.33987	30.54788
C	10.36141	6.81688	28.04950	N	12.09104	2.15405	31.86607	C	14.50513	2.35149	29.19858
H	10.50209	7.71511	27.46718	C	13.12093	2.79331	32.64316	H	15.54648	2.16441	29.48021
C	10.27817	5.59768	27.40740	C	14.16692	3.19685	31.56881	H	14.11777	1.48997	28.66107
H	10.31659	5.56220	26.32661	N	13.73303	2.49650	30.40095	C	14.97645	5.49612	30.83158
C	10.12506	4.40695	28.12103	C	12.50074	1.88909	30.56588	H	14.87549	6.48789	31.26317
C	10.09727	4.49273	29.51412	H	13.51720	2.10593	33.39747	H	15.99707	5.13378	30.99198
H	9.97097	3.58620	30.08951	H	15.19017	2.90800	31.82930	C	10.98582	1.44660	32.45099
C	10.19447	5.70958	30.16580	N	12.77443	4.04605	33.25671	H	11.33159	0.89988	33.33567
H	10.12476	5.73948	31.24446	N	14.05367	4.64222	31.53844	H	10.63024	0.73744	31.70720
C	9.95679	3.10314	27.41412	C	13.32087	5.12728	32.59833	N	5.28272	7.88822	28.83345
H	10.15984	2.26213	28.06914	O	11.91436	1.21601	29.75074	C	4.24589	7.18814	28.09287
H	10.60639	3.03012	26.54522	O	13.21554	6.29513	32.92453	C	4.61998	7.49787	26.62301
H	8.93270	3.00597	27.04970	C	14.24326	4.55266	24.86265	N	5.52347	8.60493	26.73912
Pt	10.33247	12.34278	32.30258	H	15.18677	4.89259	24.42875	C	5.95270	8.80017	28.03742
H	11.75127	12.91488	31.83189	H	14.00738	3.56118	24.48575	H	3.25860	7.56405	28.37975
N	8.83996	6.46979	23.45642	C	14.15947	7.69890	26.58767	H	3.76049	7.77668	26.00466
C	8.43819	7.83486	23.30283	H	13.91966	8.56353	27.20102	N	4.27479	5.76204	28.14768
C	7.00661	7.86236	23.91223	H	15.11973	7.86945	26.08492	N	5.19041	6.24900	26.17678
N	6.83004	6.52905	24.41733	N	7.69827	2.74817	33.00888	C	4.87121	5.19829	27.02950
C	7.89061	5.69193	24.10011	C	8.30460	3.47425	34.07892	O	6.73877	9.64805	28.41156
H	8.46415	8.12383	22.24723	C	9.76508	2.95032	34.08548	O	5.03635	4.02066	26.81828
H	6.22950	8.09746	23.17766	N	9.86857	2.27902	32.81247	C	5.55400	5.99432	24.80988
N	9.15476	8.80834	24.09490	C	8.62243	2.07408	32.22894	H	5.60045	4.91404	24.69546
N	7.08524	8.91373	24.89472	H	7.77314	3.29025	35.01759	H	4.78362	6.39582	24.14148
C	8.33402	9.49127	24.96632	H	9.97273	2.25710	34.90653	C	5.96474	9.41823	25.64006
O	7.95610	4.50283	24.30443	N	8.48203	4.90170	33.87760	H	6.26846	10.38030	26.04220
O	8.64382	10.45743	25.63693	N	10.54343	4.14734	34.24215	H	5.11889	9.56020	24.96415

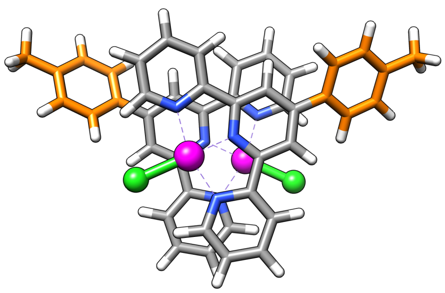

$1b_2$

C	0.50075	−0.50174	−4.63562	C	0.85586	0.55888	−2.48807	C	0.65420	−0.50536	−6.11967
C	0.14188	−0.45384	−1.83504	H	1.60740	1.31131	−4.33366	H	−0.17146	0.03528	−6.58554
C	1.03885	0.52444	−3.85625	H	−0.66088	−2.30368	−4.56263	H	0.64356	−1.51377	−6.52559
C	−0.21807	−1.50541	−3.98207	H	−0.98858	−2.26360	−2.15219	H	1.57252	−0.01486	−6.43249
C	−0.39087	−1.48849	−2.61251	H	1.31578	1.35514	−1.91892	C	−0.00138	−0.44489	−0.38362

N	-0.14578	-0.43159	2.35769	H	-0.35745	-5.46147	5.35601	C	3.25454	-2.17214	-1.41164
C	-0.00795	0.76050	0.33429	Pt	-0.07133	-0.42401	4.29570	C	3.19725	-3.45749	0.60423
C	-0.08545	-1.64399	0.34354	Cl	0.13741	-0.41410	6.60766	N	3.25185	-1.07364	0.69441
C	-0.15950	-1.62150	1.72180	C	2.43446	-6.14870	7.90297	C	3.28306	-1.02415	-0.64104
C	-0.06282	0.75065	1.71293	C	2.87858	-4.05920	6.05603	C	3.21099	-3.40384	-0.78213
H	0.04627	1.69761	-0.19295	C	3.11139	-6.37717	6.70269	H	3.18679	-4.40981	1.11231
H	-0.04351	-2.58707	-0.17737	C	1.98182	-4.85218	8.15690	H	3.32550	-0.03706	-1.07117
C	-0.03168	1.89420	2.62478	C	2.20732	-3.82449	7.26280	H	3.20328	-4.31671	-1.35987
C	-0.05020	3.88182	4.52435	C	3.32534	-5.35942	5.79453	H	3.26831	-2.08556	-2.48728
C	-0.01026	3.21835	2.22737	H	3.48839	-7.36803	6.48733	C	3.31750	0.80825	4.81318
N	-0.03525	1.57652	3.96064	H	1.44694	-4.64686	9.07457	C	3.50286	3.54371	5.01946
C	-0.04995	2.54586	4.88187	H	1.81268	-2.84233	7.48447	N	3.33744	1.58889	3.68377
C	-0.02226	4.22279	3.18262	H	3.88908	-5.57138	4.89605	C	3.36330	1.39697	6.06341
H	0.01443	3.46400	1.17769	C	2.23631	-7.24313	8.89739	C	3.45945	2.77570	6.17077
H	-0.06451	2.21450	5.90842	H	1.32863	-7.10144	9.47877	C	3.43330	2.91871	3.78770
H	-0.02004	5.25896	2.87732	H	3.06748	-7.26551	9.60425	H	3.32527	0.78222	6.94859
H	-0.07724	4.63616	5.29609	H	2.19536	-8.22001	8.42240	H	3.50976	3.23966	7.14497
C	-0.21225	-2.75568	2.64626	C	3.06392	-2.98399	5.08806	H	3.45501	3.45980	2.85464
C	-0.31950	-4.72096	4.57189	N	3.28460	-0.94730	3.26074	H	3.59442	4.61846	5.06317
N	-0.19200	-2.42439	3.97930	C	3.09331	-3.24798	3.70866	Pt	3.26204	0.50235	1.97235
C	-0.28562	-4.08379	2.26715	C	3.16486	-1.64775	5.50332	Cl	3.11464	2.23860	0.43866
C	-0.33426	-5.07707	3.23463	C	3.25654	-0.63339	4.57263				
C	-0.25661	-3.38150	4.91022	C	3.20781	-2.21363	2.80155				
H	-0.31954	-4.34194	1.21956	H	2.97839	-4.26103	3.35746				
H	-0.39835	-6.11527	2.94271	H	3.15396	-1.41175	6.55365				
H	-0.25038	-3.04064	5.93239	C	3.21548	-2.28591	1.33930				

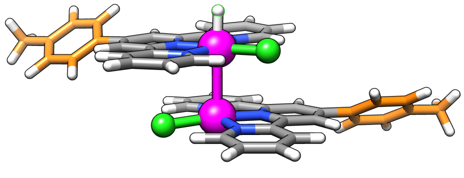

$1b_2$-H

Pt	4.30153	22.94306	17.99339	C	4.66465	29.87352	18.64553	N	7.41203	21.37230	18.17817
Cl	4.04539	20.68859	17.47572	H	5.00318	29.59792	17.65578	C	7.96301	22.79715	19.95637
N	4.70396	22.76140	19.97492	C	4.66736	31.20677	19.00474	C	8.41341	23.15010	21.21935
C	4.86154	21.62402	20.66435	H	4.97805	31.94931	18.28222	H	8.68265	22.37697	21.92260
H	4.75781	20.71550	20.09203	C	4.28235	31.61432	20.28268	C	8.52741	24.48201	21.57045
C	5.11414	21.62778	22.02137	C	3.90850	30.62588	21.19598	H	8.88983	24.75542	22.55039
H	5.21483	20.68738	22.54215	H	3.59419	30.91427	22.19018	C	8.18799	25.46119	20.64207
C	5.21095	22.83956	22.68963	C	3.91724	29.29045	20.85028	H	8.27271	26.51187	20.87499
H	5.39394	22.86935	23.75373	H	3.57752	28.56154	21.57369	C	7.75864	25.08271	19.38950
C	5.05104	24.01713	21.97848	C	4.24080	33.05840	20.65599	H	7.50717	25.79267	18.61751
H	5.11286	24.96915	22.48285	H	3.22584	33.44685	20.55625	N	7.64353	23.79044	19.04995
C	4.79551	23.97699	20.61906	H	4.88130	33.66036	20.01728	C	7.96296	17.76662	20.24481
C	4.56961	25.14492	19.77563	H	4.53950	33.21486	21.69010	C	8.88999	17.63941	21.28711
C	4.57320	26.47630	20.13948	Cl	6.85946	25.07666	16.07022	H	9.56151	18.45485	21.52059
H	4.81113	26.75692	21.15319	N	6.76860	21.74075	15.70300	C	9.00512	16.45792	21.98996
C	4.29489	27.47471	19.19128	C	6.47558	22.05204	14.43322	H	9.74151	16.37945	22.77866
C	4.00431	27.07015	17.87622	H	6.40797	23.10527	14.21202	C	8.19760	15.35652	21.69574
H	3.73324	27.81177	17.14193	C	6.28818	21.07326	13.48175	C	7.27291	15.48669	20.65892
C	4.01320	25.73408	17.53452	H	6.05464	21.35889	12.46738	H	6.62487	14.65482	20.41818
N	4.31019	24.82209	18.48859	C	6.42247	19.73815	13.84551	C	7.16211	16.65999	19.93933
C	3.68071	25.12440	16.25087	H	6.29267	18.95417	13.11425	H	6.40546	16.73192	19.16966
C	3.30070	25.82333	15.11825	C	6.73839	19.42088	15.15258	C	8.34086	14.07686	22.44980
H	3.25719	26.90128	15.14259	H	6.85205	18.38989	15.45012	H	7.44771	13.46239	22.37636
C	2.98085	25.13917	13.95891	C	6.90467	20.42731	16.09104	H	8.55291	14.25268	23.50209
H	2.68047	25.68181	13.07474	C	7.25023	20.21718	17.48766	H	9.17132	13.49119	22.05198
C	3.03898	23.75272	13.95215	C	7.44593	19.02795	18.15526	Pt	7.14916	23.05089	17.21581
H	2.77963	23.18394	13.07205	H	7.33677	18.09258	17.63016	H	8.64426	23.14766	16.75850
C	3.41767	23.08872	15.10120	C	7.81309	19.01922	19.51429				
H	3.46373	22.01327	15.16670	C	8.01717	20.25440	20.15111				
N	3.73192	23.75087	16.22208	H	8.28980	20.27605	21.19434				
C	4.29269	28.88446	19.56337	C	7.81604	21.43808	19.46988				

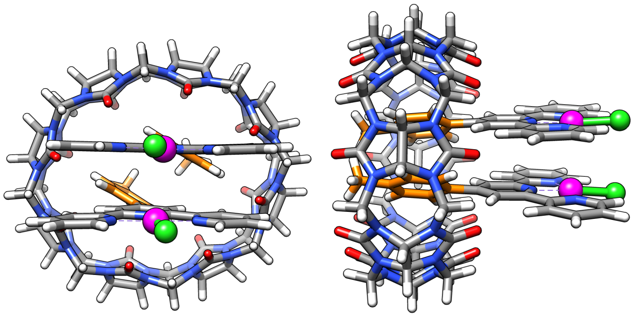

CB[8]·$1b_2$ (shorter Pt-Pt distance)

O	12.27283	4.09967	25.25387	O	13.26554	8.90344	28.98522	C	12.01030	9.07421	23.97544
C	12.90601	4.99283	25.76671	C	12.62282	6.62912	23.96447	H	12.96484	9.27832	23.48008
N	13.71314	4.89434	26.88669	H	13.51927	6.90002	23.39446	C	10.81010	9.81519	23.32385
N	13.00005	6.30121	25.31135	H	12.17774	5.73939	23.52752	H	11.09085	10.41323	22.45096
C	14.41021	6.10890	27.18333	C	13.20389	9.48828	26.17323	N	10.33796	10.67706	24.37952
H	15.49147	5.93587	27.18932	H	13.08814	10.21091	26.97550	N	12.03784	9.58953	25.32775
C	13.95020	7.07642	26.05938	H	14.08686	9.75038	25.57973	C	11.08384	10.56427	25.53492
H	14.77054	7.40425	25.41178	C	10.44557	7.49631	23.21174	O	10.93983	11.22722	26.54638
N	13.41243	8.20373	26.78021	O	9.95233	6.43452	22.91311	C	8.82886	8.93080	22.02905
N	14.01733	6.79042	28.39494	N	11.66136	7.69066	23.85067	H	9.15783	9.56331	21.19774
C	13.52142	8.05772	28.15134	N	9.92248	8.75427	22.94520	H	8.56542	7.94706	21.64933

C	9.43745	11.77364	24.15253	O	6.33527	6.84496	33.30902	C	6.18893	15.89651	31.36800
H	9.53411	12.45138	24.99512	C	6.80384	1.57628	31.82822	H	6.04264	16.95346	31.53256
H	9.74125	12.29243	23.23842	H	6.71877	0.95853	30.93798	C	6.84299	15.14700	32.32825
C	6.44501	8.78496	22.62367	H	6.48933	0.99659	32.70226	H	7.22272	15.56727	33.24710
O	6.30330	7.61057	22.37943	C	7.40732	4.29675	34.10858	N	7.03553	13.83260	32.17407
N	7.63769	9.49428	22.60563	H	7.66523	5.25051	34.55868	C	6.66596	8.59599	28.98346
N	5.44406	9.68943	22.93672	H	7.06606	3.61191	34.89091	C	6.66656	9.04204	27.65717
C	7.45106	10.89866	22.83062	C	9.13641	1.50160	31.04511	H	6.84583	10.08489	27.43596
H	7.79155	11.47803	21.96702	O	8.91384	1.00985	29.96457	C	6.47494	8.15901	26.61099
C	5.92324	11.02869	23.09500	N	8.19228	1.92457	31.97020	H	6.49613	8.52914	25.59449
H	5.41809	11.70438	22.39881	N	10.38083	1.71504	31.61427	C	6.26869	6.79752	26.83510
N	5.86315	11.57353	24.43421	C	8.78081	2.35982	33.20356	C	6.23598	6.36166	28.16214
N	8.04623	11.42340	24.03921	H	8.43225	1.74362	34.03791	H	6.05372	5.31523	28.36837
C	7.10781	11.84124	24.95436	C	10.31025	2.23720	32.94506	C	6.44177	7.23399	29.21155
O	7.33470	12.38535	26.02285	H	10.81759	1.57093	33.64875	H	6.39146	6.85723	30.22283
C	4.05657	9.31491	22.95500	N	10.78721	3.59113	33.12482	C	6.12618	5.83200	25.70699
H	3.97920	8.35029	22.45999	N	8.60599	3.75981	33.52089	H	7.06074	5.28856	25.55780
H	3.48108	10.05694	22.39519	C	9.79017	4.45990	33.50158	H	5.88790	6.32364	24.76998
C	4.65888	12.08881	25.03659	O	9.93909	5.63396	33.79928	H	5.35320	5.09506	25.91027
H	4.06692	12.58331	24.26314	C	11.59563	1.29772	30.96939	Pt	10.98227	12.89002	32.25113
H	4.95694	12.81867	25.78300	H	12.21297	0.75622	31.69106	Cl	11.36819	14.91873	33.28745
C	3.08220	7.91801	24.72784	H	11.31478	0.62779	30.16080	N	11.84815	11.64571	33.59716
O	3.28526	6.85444	24.19341	C	12.18160	3.94588	33.21366	C	12.40193	11.98916	34.76492
N	3.49120	9.16282	24.26906	H	12.24525	4.86782	33.78356	H	12.34251	13.03710	35.01661
N	2.35487	8.13440	25.88526	H	12.70898	3.15283	33.74855	C	13.01480	11.05108	35.57486
C	2.91503	10.24144	25.02149	C	12.52596	2.45688	29.01132	H	13.46058	11.36803	36.50572
H	2.24209	10.83140	24.39161	O	11.94802	1.80358	28.17614	C	13.05494	9.72813	35.16572
C	2.18595	9.52128	26.19107	N	12.36502	2.36465	30.38707	H	13.53999	8.97986	35.77611
H	1.12283	9.77310	26.25656	N	13.51024	3.40245	28.78343	C	12.46823	9.36621	33.96381
N	2.87360	10.01320	27.37009	C	13.34352	3.13044	31.10673	H	12.48680	8.34220	33.62354
N	3.83877	11.11588	25.69903	H	14.00014	2.47003	31.68155	C	11.86172	10.33575	33.18369
C	3.77215	11.00951	27.06885	C	14.08789	3.91316	29.98802	C	11.21732	10.08082	31.89608
O	4.37023	11.71253	27.86767	H	15.17003	3.74960	29.99411	C	11.04737	8.87295	31.25333
C	1.63533	7.07078	26.52768	N	13.80575	5.30088	30.30392	H	11.44806	7.97330	31.68900
H	1.69294	6.20533	25.87297	N	12.83833	4.17670	31.95934	C	10.41613	8.82402	30.00281
H	0.58907	7.36783	26.64544	C	13.15262	5.43691	31.50625	C	9.99926	10.03066	29.42209
C	2.37499	9.82322	28.70965	O	12.93144	6.48191	32.09699	H	9.51713	10.03068	28.45964
H	2.83941	10.57788	29.33763	C	14.04621	3.62534	27.47014	C	10.17727	11.22310	30.09509
H	1.29063	9.97648	28.70676	H	15.13500	3.52594	27.50783	N	10.75644	11.20638	31.31380
C	2.71056	5.42315	28.01103	H	13.63600	2.85484	26.82282	C	9.78707	12.56951	29.67796
O	2.97392	4.60386	27.16171	C	14.50186	6.40578	29.69235	C	9.14710	12.87294	28.49198
N	2.15453	6.67373	27.80626	H	15.56450	6.15287	29.61490	H	8.91039	12.09807	27.78498
N	2.86282	5.26485	29.38188	H	14.38692	7.26347	30.34859	C	8.82595	14.18760	28.20213
C	1.86875	7.36628	29.02614	Pt	7.89716	12.56696	33.50334	H	8.32018	14.41134	27.27546
H	0.80455	7.61850	29.08068	Cl	8.27585	14.03015	35.25124	C	9.15280	15.18446	29.10613
C	2.31943	6.36701	30.12604	N	8.56901	10.86953	34.37434	H	8.92503	16.22185	28.91220
H	1.49544	6.02853	30.76338	C	9.12657	10.76147	35.58497	C	9.78356	14.84110	30.28606
N	3.26549	7.12512	30.90661	H	9.20125	11.68202	36.14334	H	10.06576	15.56235	31.03767
N	2.66441	8.54205	29.29357	C	9.56826	9.54500	36.06771	N	10.08857	13.57022	30.56885
C	3.40664	8.42457	30.45400	H	10.01051	9.49701	37.05148	C	10.17976	7.54204	29.34172
O	4.03567	9.30943	30.99925	C	9.43535	8.41723	35.27512	C	10.08647	7.44305	27.94956
C	3.02813	3.95451	29.94715	H	9.78136	7.45087	35.60810	H	10.24620	8.31673	27.33458
H	2.13534	3.69735	30.52905	C	8.85280	8.52805	34.02466	C	9.83727	6.23096	27.33804
H	3.12857	3.25611	29.12073	H	8.73219	7.65186	33.41327	H	9.78150	6.18144	26.25862
C	3.66752	6.74041	32.23041	C	8.41205	9.75959	33.58099	C	9.64527	5.06491	28.08294
H	4.12664	7.60810	32.69479	C	7.78009	10.00253	32.28451	C	9.75554	5.16332	29.47056
H	2.77913	6.45474	32.80471	C	7.49911	9.09417	31.28335	H	9.62080	4.27593	30.07482
C	5.14898	2.83901	30.50874	H	7.75573	8.05774	31.41908	C	10.01985	6.37034	30.08991
O	5.27587	2.22278	29.47725	C	6.91274	9.52515	30.08443	H	10.07467	6.40451	31.16908
N	4.18759	3.80144	30.78191	C	6.58835	10.88279	29.95518	C	9.29553	3.77655	27.41729
N	5.91161	2.69060	31.65889	H	6.06683	11.22959	29.07902	H	9.51495	2.91666	28.04118
C	4.23988	4.27228	32.13413	C	6.87427	11.76317	30.97715	H	9.82610	3.65810	26.47618
H	3.29001	4.08583	32.64522	N	7.48024	11.30393	32.09072	H	8.22725	3.74705	27.19532
C	5.43192	3.48835	32.74939	C	6.59744	13.19781	31.03708				
H	5.14490	2.85421	33.59429	C	5.93512	13.91162	30.05319				
N	6.32109	4.53226	33.19776	H	5.58166	13.40136	29.17028				
N	4.62140	5.65860	32.29576	C	5.72366	15.27036	30.22318				
C	5.82038	5.79675	32.96334	H	5.19799	15.83341	29.46528				

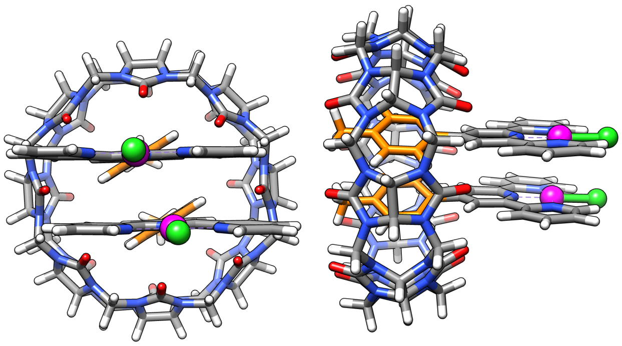

CB[8]·$1b_2$ (longer Pt-Pt distance)

C	5.77954	12.13131	19.91108	H	7.47966	14.94113	15.63875	H	13.13316	13.68733	19.57565
O	6.01035	11.79680	21.04923	H	7.83157	13.20233	15.63956	C	11.52979	16.52313	17.88536
N	4.52984	12.33941	19.34976	C	10.01690	12.94906	19.97901	H	11.10092	17.35749	17.33802
N	6.71594	12.34169	18.90509	O	9.93571	12.46639	21.08354	H	12.43431	16.18286	17.37081
C	4.59631	12.73725	17.98038	N	9.09042	12.81836	18.95100	C	12.55730	15.40559	22.14607
H	3.93810	12.11812	17.36354	N	11.07253	13.68987	19.47815	O	12.37964	14.57508	23.00643
C	6.09860	12.58321	17.62793	C	9.59858	13.31669	17.69962	N	12.42243	15.22208	20.78005
H	6.30144	11.75975	16.93680	H	9.78429	12.49312	17.00254	N	12.98459	16.70947	22.33816
N	6.42526	13.83984	17.00429	C	10.88997	14.07078	18.11471	C	12.87908	16.35333	20.02015
N	4.34041	14.14329	17.72092	H	11.76458	13.79280	17.51851	H	13.75309	16.08218	19.41895
C	5.40171	14.75463	17.08246	N	10.55063	15.46212	17.86961	C	13.19182	17.41559	21.10969
O	5.41200	15.89161	16.63830	N	8.81151	14.33475	17.05888	H	14.21606	17.79903	21.05814
C	8.05526	11.82579	19.03208	C	9.38712	15.58472	17.14357	N	12.25353	18.47556	20.81720
H	8.21787	11.06414	18.26049	O	8.95728	16.60072	16.62697	N	11.89550	16.99859	19.18937
H	8.13292	11.36197	20.01203	C	12.27931	13.90420	20.22442	C	11.56351	18.26214	19.63578
C	7.64241	14.07378	16.27039	H	12.27654	13.20830	21.05903	O	10.83427	19.05670	19.07714

C	13.43997	17.17021	23.61898
H	13.48471	16.30408	24.27357
H	14.44056	17.59723	23.51209
C	12.34081	19.77932	21.40851
H	11.78118	20.45995	20.77331
H	13.38968	20.09435	21.43463
C	11.78766	17.81946	25.33819
O	11.64878	16.72735	25.83395
N	12.59257	18.13965	24.25811
N	11.19963	19.00569	25.76999
C	12.56044	19.52763	23.92786
H	13.57446	19.93053	23.84913
C	11.73603	20.15355	25.08431
H	12.33722	20.76688	25.76258
N	10.77407	20.97419	24.39813
N	11.79592	19.88704	22.74426
C	10.80184	20.79788	23.03386
O	10.09198	21.37215	22.22466
C	10.64063	19.08548	27.09636
H	10.65988	18.07901	27.50574
H	11.26272	19.73880	27.71682
C	9.96500	21.97139	25.03630
H	10.59711	22.53127	25.73251
H	9.60278	22.64335	24.26464
C	8.22216	18.71360	27.45403
O	8.26538	17.51144	27.56989
N	9.28540	19.55272	27.16109
N	7.10435	19.52158	27.62908
C	8.90308	20.92614	27.08424
H	9.56107	21.54769	27.69891
C	7.42911	20.92211	27.56628
H	7.28878	21.39738	28.54158
N	6.74357	21.67661	26.54883
N	8.80893	21.47847	25.74410
C	7.54934	21.97797	25.47610
O	7.22382	22.60418	24.48016
C	5.94483	19.02641	28.32644
H	5.81549	19.59837	29.25269
H	6.13537	17.98543	28.57442
C	5.39724	22.16843	26.69084
H	5.26385	22.96457	25.96566
H	5.29020	22.57949	27.69720
C	3.98432	17.90424	27.37567
O	4.35386	16.77344	27.58626
N	4.72655	19.06399	27.56653
N	2.72684	18.29573	26.95210
C	2.58788	19.71276	26.85071
H	1.70189	20.05136	27.39667
C	3.91782	20.24883	27.44480
H	3.79203	20.73279	28.41878
N	2.58040	20.26884	25.50855
N	4.35200	21.21186	26.46990
C	3.55124	21.23192	25.34790
O	3.66443	21.99874	24.40819
C	1.64848	17.36305	26.78992
H	0.76073	17.76786	27.28501
H	1.94011	16.43646	27.27694
C	1.41781	20.23915	24.65302
H	1.56240	21.00476	23.89635
H	0.53075	20.48330	25.24624
C	1.39241	15.72713	24.97820
O	1.88433	14.78820	25.55940
N	1.32931	17.03592	25.42698
N	0.73916	15.68700	23.75684
C	0.18295	16.95155	23.37961
H	-0.89222	16.85623	23.19625
C	0.52043	17.87327	24.58366
H	-0.36683	18.21754	25.12512
N	0.81616	17.61890	22.26463
N	1.18826	18.99473	23.97568
C	1.32172	18.86148	22.60809
O	1.76252	19.69392	21.84134
C	0.42106	14.44330	23.11462
H	-0.65005	14.41400	22.89904
H	0.67096	13.64922	23.81303
C	0.56082	17.26647	20.89805
H	-0.51075	17.07973	20.76728
H	0.85046	18.11607	20.28625
C	2.16909	13.25285	21.83654
O	2.61536	12.60907	22.75533
N	1.14191	14.17894	21.89931
N	2.56268	13.17518	20.50293
C	0.82799	14.75340	20.63133
H	-0.24605	14.69119	20.43297
C	1.68763	13.92592	19.63888
H	1.09611	13.25668	19.00656
N	2.32352	14.93330	18.83272
N	1.28994	16.11500	20.41285
C	2.10411	16.20723	19.30355
O	2.53049	17.23617	18.80562
C	3.31232	12.03250	20.04433
H	2.68350	11.42157	19.38851
H	3.57520	11.45543	20.92686
C	3.00239	14.66775	17.59794
H	3.07688	15.60526	17.05598
H	2.39607	13.96487	17.01824
Pt	4.21945	23.48122	18.53314
Cl	3.89970	25.30866	17.15655
N	3.86779	21.97950	17.22015
C	3.59093	22.11007	15.91952
H	3.55079	23.12314	15.55096
C	3.39071	21.00487	15.11338
H	3.16350	21.14934	14.06804
C	3.48581	19.73976	15.66705
H	3.33764	18.85916	15.05930
C	3.78688	19.60245	17.01272
H	3.85835	18.62448	17.46006
C	3.97499	20.73272	17.78727
C	4.32143	20.72212	19.20934
C	4.49739	19.63540	20.04077
H	4.35091	18.64226	19.65163
C	4.85505	19.82369	21.38577
C	5.04391	21.13612	21.84190
H	5.28557	21.32563	22.87256
C	4.83028	22.20314	20.99316
N	4.48831	21.96406	19.71025
C	4.88932	23.63227	21.30360
C	5.17055	24.15474	22.55299
H	5.38518	23.49007	23.37168
C	5.17538	25.52761	22.73360
H	5.39154	25.94083	23.70847
C	4.89406	26.35971	21.66249
H	4.87763	27.43413	21.76874
C	4.62146	25.79756	20.42996
H	4.39314	26.38331	19.55272
N	4.62037	24.47187	20.25143
C	4.99785	18.68487	22.29004
C	5.83661	18.72872	23.40745
H	6.43427	19.60924	23.60240
C	5.96673	17.63279	24.24152
H	6.64790	17.67798	25.08020
C	5.25911	16.45328	24.01002
C	4.39953	16.42393	22.90923
H	3.82912	15.52628	22.71101
C	4.28068	17.50464	22.06302
H	3.59739	17.44881	21.23116
C	5.41465	15.25543	24.88440
H	4.48190	15.01973	25.39319
H	6.17485	15.40672	25.64401
H	5.67873	14.37935	24.29317
Pt	7.16655	22.39094	16.55059
Cl	6.90838	24.10740	15.02579
N	6.85750	20.83111	15.29856
C	6.61924	20.89471	13.98375
H	6.58517	21.88963	13.56679
C	6.43672	19.75298	13.22712
H	6.25413	19.84495	12.16686
C	6.49864	18.51701	13.84955
H	6.35893	17.60819	13.28174
C	6.74959	18.44780	15.20958
H	6.80201	17.49789	15.71264
C	6.93119	19.61444	15.92999
C	7.22457	19.67114	17.36294
C	7.37544	18.62045	18.24515
H	7.27416	17.61275	17.88311
C	7.75124	18.86411	19.57394
C	7.91049	20.19834	19.98268
H	8.18992	20.42610	20.99724
C	7.72178	21.22808	19.08423
N	7.39241	20.93306	17.80919
C	7.82157	22.66937	19.31859
C	8.09647	23.25218	20.54233
H	8.29181	22.63283	21.40239
C	8.13986	24.63331	20.64616
H	8.35455	25.09171	21.60040
C	7.89437	25.41086	19.52766
H	7.91419	26.48920	19.57269
C	7.61791	24.79072	18.32330
H	7.39865	25.33416	17.41755
N	7.58910	23.45903	18.21879
C	8.00382	17.76231	20.49984
C	8.90386	17.92055	21.56002
H	9.43170	18.85354	21.67516
C	9.16062	16.88984	22.43731
H	9.86493	17.03995	23.24462
C	8.51479	15.65682	22.31953
C	7.62430	15.49642	21.25787
H	7.10236	14.55535	21.15049
C	7.37661	16.52088	20.36187
H	6.64948	16.36517	19.57591
C	8.76668	14.57025	23.30910
H	8.13291	13.70762	23.13063
H	8.59575	14.92732	24.32405
H	9.80110	14.23470	23.26590

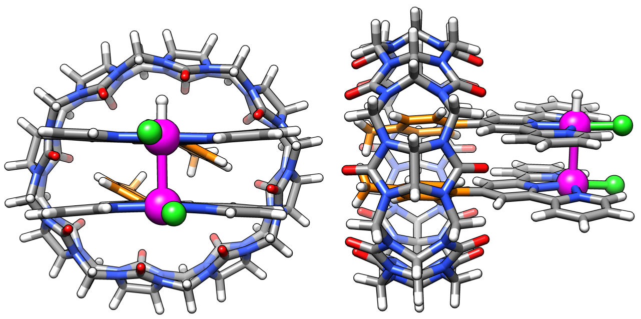

CB[8]·1b$_2$-H

O	12.15430	4.12119	25.22314
C	12.81336	5.00541	25.71827
N	13.63129	4.90116	26.82983
N	12.93105	6.30680	25.24767
C	14.35034	6.10554	27.11215
H	15.42821	5.91251	27.12187
C	13.90972	7.06665	25.97566
H	14.73377	7.36000	25.31629
N	13.41042	8.21943	26.68124
N	13.96841	6.80964	28.31571
C	13.50542	8.08662	28.05499
O	13.26388	8.94791	28.87712
C	12.54603	6.62761	23.90140
H	13.44222	6.86980	23.31764
H	12.07480	5.74371	23.48013
C	13.24009	9.49936	26.05410
H	13.15207	10.23849	26.84480
H	14.12693	9.72208	25.45118
C	10.40172	7.54135	23.11782
O	9.88648	6.48990	22.82070
N	11.60713	7.71063	23.78674
N	9.92339	8.80863	22.81977
C	12.00267	9.08682	23.87488
H	12.95180	9.25060	23.35488
C	10.81150	9.85233	23.23420
H	11.10211	10.47357	22.38112
N	10.33648	10.68899	24.31214
N	12.07499	9.62627	25.21339
C	11.11364	10.58349	25.44773
O	10.98742	11.24159	26.46510
C	8.80960	9.01124	21.93486
H	9.12030	9.67174	21.11928
H	8.54148	8.03945	21.52860
C	9.44345	11.79644	24.10433
H	9.55941	12.46793	24.94945
H	9.73818	12.31846	23.18897
C	6.44300	8.82713	22.59035
O	6.30692	7.65758	22.32195
N	7.62689	9.55116	22.55231
N	5.44179	9.71199	22.95627
C	7.42995	10.94891	22.80851
H	7.74092	11.54793	21.94721
C	5.90831	11.05609	23.11545
H	5.37564	11.73196	22.44020
N	5.87763	11.58599	24.46112
N	8.04946	11.45887	24.01037
C	7.13175	11.84792	24.95877
O	7.38238	12.36735	26.03430
C	4.05561	9.32886	22.96887
H	3.98203	8.37490	22.45308
H	3.47500	10.08015	22.42698
C	4.68235	12.05724	25.11318
H	4.06085	12.56774	24.37339
H	4.98840	12.76623	25.87639
C	3.12302	7.88748	24.73139
O	3.32333	6.83598	24.17232
N	3.49322	9.14489	24.27835
N	2.43676	8.07349	25.91987
C	2.94953	10.20387	25.07771
H	2.27121	10.82319	24.48232
C	2.23637	9.45504	26.23896
H	1.16728	9.68109	26.30496
N	2.90964	9.94851	27.42290
N	3.90000	11.04511	25.76277
C	3.81814	10.93953	27.13328
O	4.41151	11.63779	27.93889
C	1.70886	6.99752	26.53256
H	1.78641	6.14073	25.86859
H	0.65766	7.28542	26.63180

C	2.41333	9.73681	28.75867	H	12.73116	3.25649	33.71367	H	5.99672	5.32849	28.41023
H	2.87826	10.48139	29.39819	C	12.49438	2.44753	29.01117	C	6.41431	7.24092	29.25260
H	1.32882	9.88996	28.75901	O	11.91677	1.76653	28.19713	H	6.33875	6.86872	30.26405
C	2.76698	5.34320	28.01987	N	12.38896	2.34846	30.38950	C	6.13027	5.83137	25.74827
O	3.05118	4.53294	27.16805	N	13.42259	3.43994	28.74710	H	7.03570	5.22974	25.65501
N	2.20254	6.58926	27.81658	C	13.33012	3.18170	31.07901	H	5.97033	6.32674	24.79638
N	2.90122	5.17557	29.39118	H	14.01370	2.57211	31.67822	H	5.30514	5.14359	25.91607
C	1.90345	7.27577	29.03625	C	14.04173	3.95479	29.93223	Cl	11.18119	15.03415	33.30978
H	0.84006	7.53391	29.07698	H	15.12269	3.78464	29.90382	N	11.59032	11.69401	33.70171
C	2.33214	6.26612	30.13518	N	13.77769	5.34364	30.24622	C	12.07374	12.03838	34.90263
H	1.49465	5.91493	30.74761	N	12.77853	4.23665	31.89388	H	11.97108	13.07959	35.16667
N	3.25344	7.01971	30.94652	C	13.10751	5.49323	31.43770	C	12.66156	11.10410	35.72812
N	2.70286	8.44567	29.32295	O	12.88458	6.54370	32.01701	H	13.04256	11.41655	36.68858
C	3.41764	8.31910	30.50041	C	13.94473	3.63290	27.42271	C	12.76411	9.78429	35.29988
O	4.04260	9.19596	31.06226	H	15.03216	3.50959	27.44355	H	13.23856	9.04202	35.92507
C	3.07549	3.86247	29.94733	H	13.50713	2.86324	26.79250	C	12.24843	9.42393	34.06969
H	2.17697	3.58728	30.51229	C	14.46381	6.44188	29.61510	H	12.31223	8.40575	33.71656
H	3.19866	3.17363	29.11597	H	15.52651	6.19044	29.53259	C	11.65122	10.38517	33.26680
C	3.62186	6.62410	32.27628	H	14.35158	7.30774	30.26096	C	11.08423	10.13409	31.95403
H	4.05948	7.49070	32.76299	Pt	8.14151	12.55950	33.48093	C	10.96081	8.93166	31.28910
H	2.72074	6.32285	32.82133	Cl	8.51102	13.98658	35.27034	H	11.33812	8.03054	31.74225
C	5.19094	2.75490	30.54064	N	8.61609	10.83455	34.41977	C	10.39422	8.88505	30.00712
O	5.34460	2.15115	29.50539	C	9.08312	10.70499	35.67029	C	10.01350	10.09581	29.40277
N	4.22440	3.71774	30.79926	H	9.19390	11.62636	36.21988	H	9.59177	10.09816	28.41242
N	5.91986	2.58956	31.70894	C	9.38781	9.46992	36.19853	C	10.14668	11.28887	30.08064
C	4.23623	4.16294	32.16267	H	9.75780	9.40700	37.21089	N	10.64066	11.25637	31.34347
H	3.27976	3.94551	32.64845	C	9.22414	8.33800	35.41197	C	9.81159	12.62893	29.63384
C	5.43227	3.39353	32.78942	H	9.47861	7.35648	35.78119	C	9.29262	12.94624	28.38982
H	5.14778	2.76604	33.64000	C	8.72686	8.46811	34.12931	H	9.08953	12.16873	27.67601
N	6.31301	4.44975	33.23044	H	8.57590	7.59282	33.52421	C	9.04929	14.26317	28.05835
N	4.58375	5.55190	32.35806	C	8.40755	9.72011	33.63748	H	8.63193	14.49447	27.09049
C	5.78503	5.70670	33.01351	C	7.82761	9.97874	32.32460	C	9.34390	15.26691	28.97590
O	6.28152	6.76181	33.36492	C	7.49433	9.07537	31.33657	H	9.17743	16.30889	28.74787
C	6.84540	1.50370	31.87769	H	7.68146	8.02706	31.49427	C	9.84445	14.92344	30.20940
H	6.77131	0.87773	30.99223	C	6.94692	9.52182	30.12265	H	10.08939	15.64627	30.97137
H	6.55341	0.92186	32.75779	C	6.69696	10.89308	29.97288	N	10.05598	13.63999	30.54079
C	7.39488	4.22957	34.15137	H	6.19897	11.25388	29.08891	C	10.17182	7.60475	29.33935
H	7.62977	5.18588	34.60842	C	7.03765	11.77465	30.97766	C	10.10201	7.50747	27.94642
H	7.05971	3.53444	34.92710	N	7.61913	11.29651	32.09746	H	10.28198	8.37928	27.33439
C	9.17410	1.46928	31.08278	C	6.84248	13.21970	31.01657	C	9.84368	6.29964	27.32790
O	8.95517	0.95985	30.01031	C	6.19521	13.95370	30.03647	H	9.80017	6.25508	26.24797
N	8.22596	1.89151	32.00617	H	5.80120	13.44844	29.16764	C	9.62332	5.13606	28.06576
N	10.41765	1.70748	31.64557	C	6.04449	15.32056	30.19505	C	9.71762	5.22963	29.45633
C	8.81375	2.32840	33.24077	H	5.52753	15.89787	29.44218	H	9.56029	4.34258	30.05661
H	8.48705	1.69489	34.07097	C	6.56557	15.93958	31.32203	C	9.98451	6.43127	30.08195
C	10.34345	2.24579	32.96921	H	6.46928	17.00380	31.47611	H	10.01863	6.46121	31.16196
H	10.87620	1.60419	33.67702	C	7.20794	15.17547	32.27678	C	9.25545	3.85194	27.40114
N	10.78159	3.61595	33.12622	H	7.62985	15.59093	33.17908	H	9.74338	2.99707	27.86027
N	8.60797	3.71886	33.57375	N	7.33302	13.85006	32.13895	H	9.51304	3.85885	26.34633
C	9.76694	4.45733	33.51648	C	6.67823	8.59602	29.02427	H	8.18096	3.67856	27.48387
O	9.88472	5.63895	33.79742	C	6.71166	9.03533	27.69589	Pt	10.85451	12.95970	32.28092
C	11.63633	1.29037	31.00643	H	6.92375	10.07125	27.47186	H	12.29477	13.18583	31.71634
H	12.26467	0.78009	31.74069	C	6.51347	8.15372	26.64984				
H	11.36279	0.59324	30.21871	H	6.56273	8.51886	25.63224				
C	12.16420	4.01761	33.17177	C	6.27214	6.79865	26.87483				
H	12.21057	4.95591	33.71623	C	6.20557	6.36967	28.20311				

11. Frontier orbitals selection for assembly CB[8]·$\mathbf{1b_2}$

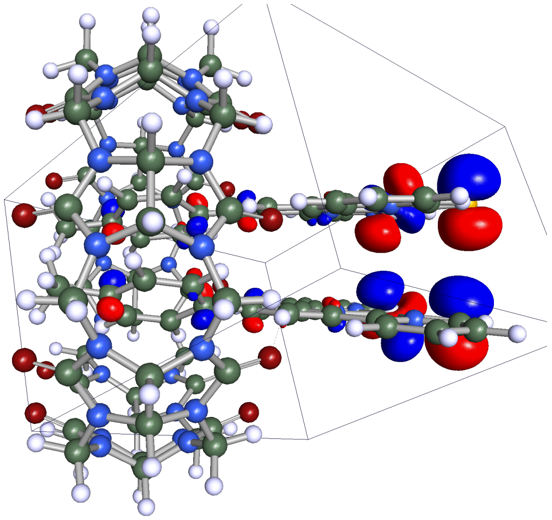

Figure S6. HOMO of complex CB[8]·**1b**$_2$ (shorter Pt-Pt distance conformation).

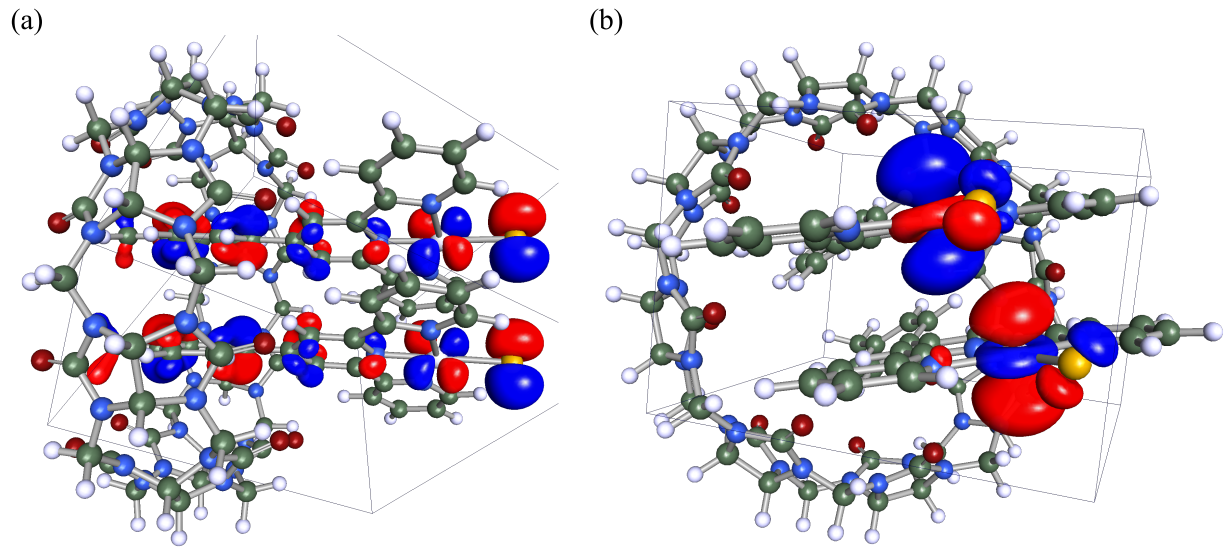

Figure S7. (a) HOMO and (b) HOMO-5 of complex CB[8]·**1b**$_2$ (longer Pt-Pt distance conformation).

12. References

1 F. Diederich, P. J. Stang and R. R. Tykwinski, in *Modern supramolecular chemistry*, Wiley-VCH Verlag GmbH & Co. KGaA, 2008.

2 W. L. F. Armarego and C. L. L. Chai, *Purification of Laboratory Chemicals*, Butterworth-Heinemann, 2012.

3 K. Kotturi and E. Masson, *Chem. - Eur. J.*, 2018, **24**, 8670–8678.

4 H. Barbero, N. A. Thompson and E. Masson, *J. Am. Chem. Soc.*, 2020, **142**, 867–873.

5 Y. Ji, D. A. DiRocco, C. M. Hong, M. K. Wismer and M. Reibarkh, *Org. Lett.*, 2018, **20**, 2156–2159.

6 N. A. Till, L. Tian, Z. Dong, G. D. Scholes and D. W. C. MacMillan, *J. Am. Chem. Soc.*, 2020, **142**, 15830–15841.

7 S. Grimme, C. Bannwarth and P. Shushkov, *J. Chem. Theory Comput.*, 2017, **13**, 1989–2009.

8 C. Bannwarth, S. Ehlert and S. Grimme, *J. Chem. Theory Comput.*, 2019, **15**, 1652–1671.

9 M. Brieg, J. Setzler, S. Albert and W. Wenzel, *Phys. Chem. Chem. Phys.*, 2017, **19**, 1677–1685.

10 A. V. Onufriev and D. A. Case, *Annu. Rev. Biophys.*, 2019, **48**, 275–296.

11 *TURBOMOLE V7.2.1 2015, a development of University of Karlsruhe and Forschungszentrum Karlsruhe GmbH, 1989-2007, TURBOMOLE GmbH, since 2007; available from http://www.turbomole.com*.

12 R. Ahlrichs, M. Bär, M. Häser, H. Horn and C. Kölmel, *Chem. Phys. Lett.*, 1989, **162**, 165–169.

13 M. Sierka, A. Hogekamp and R. Ahlrichs, *J. Chem. Phys.*, 2003, **118**, 9136–9148.

14 P. Deglmann, K. May, F. Furche and R. Ahlrichs, *Chem. Phys. Lett.*, 2004, **384**, 103–107.

15 A. Klamt and G. Schüürmann, *J. Chem. Soc. Perkin Trans. 2*, 1993, 799–805.

16 A. Klamt, *J. Phys. Chem.*, 1995, **99**, 2224–2235.
